# Is It First the Egg or the Shrimp? – Diversity and Variation in Microbial Communities Colonizing Broods of the Vent Shrimp *Rimicaris exoculata* During Embryonic Development

**DOI:** 10.3389/fmicb.2019.00808

**Published:** 2019-04-17

**Authors:** Pierre Methou, Ivan Hernández-Ávila, Johanne Aube, Valérie Cueff-Gauchard, Nicolas Gayet, Louis Amand, Bruce Shillito, Florence Pradillon, Marie-Anne Cambon-Bonavita

**Affiliations:** ^1^Univ Brest, CNRS, Ifremer, Laboratoire de Microbiologie des Environnements Extrêmes, Plouzané, France; ^2^Ifremer, Laboratoire Environnement Profond (REM/EEP/LEP), Plouzané, France; ^3^Unité Biologie des Organismes et Ecosystèmes Aquatiques, Muséum National d’Histoire Naturelle, Eq. Adaptations aux Milieux Extrêmes (BOREA), CNRS, IRD, Sorbonne Université, Université de Caen Normandie, Université des Antilles, Paris, France

**Keywords:** hydrothermal, shrimp, microbial colonization, Alvinocarididae, egg development

## Abstract

*Rimicaris exoculata* is one of the most well-known and emblematic species of endemic vent fauna. Like many other species from these ecosystems, *Rimicaris* shrimps host important communities of chemosynthetic bacteria living in symbiosis with their host inside the cephalothorax and gut. For many of these symbiotic partners, the mode of transmission remains to be elucidated and the starting point of the symbiotic relationship is not yet defined, but could begin with the egg. In this study, we explored the proliferation of microbial communities on *R. exoculata* broods through embryonic development using a combination of NGS sequencing and microscopy approaches. Variations in abundance and diversity of egg microbial communities were analyzed in broods at different developmental stages and collected from mothers at two distinct vent fields on the Mid-Atlantic Ridge (TAG and Snake Pit). We also assessed the specificity of the egg microbiome by comparing communities developing on egg surfaces with those developing on the cuticle of pleopods, which are thought to be exposed to similar environmental conditions because the brood is held under the female’s abdomen. In terms of abundance, bacterial colonization clearly increases with both egg developmental stage and the position of the egg within the brood: those closest to the exterior having a higher bacterial coverage. Bacterial biomass increase also accompanies an increase of mineral precipitations and thus clearly relates to the degree of exposure to vent fluids. In terms of diversity, most bacterial lineages were found in all samples and were also those found in the cephalothorax of adults. However, significant variation occurs in the relative abundance of these lineages, most of this variation being explained by body surface (egg vs. pleopod), vent field, and developmental stage. The occurrence of symbiont-related lineages of *Epsilonbacteraeota, Gammaproteobacteria, Zetaproteobacteria*, and Mollicutes provide a basis for discussion on both the acquisition of symbionts and the potential roles of these bacterial communities during egg development.

## Introduction

Deep-sea hydrothermal vents are oases of life, mainly sustained by primary microbial chemosynthetic production. In these ecosystems, microbial life thrives in several microhabitats from water fluids to rock or sediment surfaces (Campbell et al., 2006). As in other aquatic environments, the bodies of vent fauna also offer suitable surfaces for bacterial communities to establish themselves and have never been observed without microorganisms (López-García et al., 2002; Duperron et al., 2009; Goffredi, 2010). More intricate and specific, relationships formed by chemosynthetic symbioses, where symbionts provide most of their host’s nutrition, are also widespread in several dominant vent megafauna taxa (Dubilier et al., 2008). Among these, the alvinocaridid shrimp *Rimicaris exoculata* (Williams and Rona, 1986) is found in several vent fields on the Mid-Atlantic Ridge (MAR), like TAG and Snake Pit, where it forms dense aggregates of thousands of shrimps per m^2^ close to hydrothermal vents (Segonzac et al., 1993; Copley et al., 2007).

Adult stages of *R. exoculata* host episymbiotic bacteria in their cephalothorax (or branchial chamber) and on mouthparts, which are modified and adapted to the colonization and growth of the symbiotic bacteria (Segonzac et al., 1993; Zbinden et al., 2004). Moreover, shrimps host a distinct epibiotic microbial community in their gut (Zbinden and Cambon-Bonavita, 2003; Durand et al., 2010, 2015). The bacterial assemblage in the cephalothorax provides nutrition to the shrimp by direct transfer of organic carbon generated by chemosynthesis (Ponsard et al., 2013). In addition, the metabolic activity of the bacteria could also protect the shrimp from harmful vent fluids by facilitating detoxification processes (Zbinden et al., 2008; Ponsard et al., 2013; Jan et al., 2014).

The microbial community hosted in the cephalothorax is particularly dynamic due to the short molting cycle of the shrimp (roughly 10 days). Because bacteria colonize the inner surface of the cephalothorax and mouthparts, these episymbionts are eliminated after each molt (Corbari et al., 2008b). The molting cycle of the shrimp generates a constant cycle of production of new body surfaces followed by symbiont recolonization, development, and accumulation of mineral deposits (Corbari et al., 2008a,b). In contrast, as the gut has no cuticle layer and is thus not subjected to surface renewal during molting, symbionts in this part of the body are supposedly maintained throughout the life of the animal following their acquisition (Durand et al., 2010).

The symbiotic bacterial assemblages are diverse in both cephalothorax and gut compartments, but are usually dominated by *Epsilonbacteraeota* and *Gammaproteobacteria* in the cephalothorax (Zbinden et al., 2008; Petersen et al., 2010; Jan et al., 2014) and by *Deferribacteres, Entomoplamatales*, and *Epsilonbacteraeota* in the gut (Durand et al., 2010, 2015; Cowart et al., 2017). Whereas *Epsilonbacteraeota-* and *Gammaproteobacteria-*related lineages have been found in abundance in the surrounding fluids (Petersen et al., 2010), *Deferribacteres* and *Entomoplamatales* symbionts have not yet been detected in the shrimp environment.

In caridean shrimps, females molt just before mating and extruding eggs. During this molt, pleopods become modified, with more developed setae, which is related to their role in holding the eggs during the brooding period (Correa and Thiel, 2003; Bauer, 2013). After mating, the eggs are extruded, mixed with sperm and covered with a mucus layer that ensures they remain adhered together and to the pleopods (Fisher and Clark, 1983). Since ovigerous females remain in dense populations crawling on the walls of active chimneys during the entire brooding period (Hernández-Ávila, 2016), their eggs experience environmental conditions found in the adult aggregations. They are thus exposed to the dynamic mixing between seawater and warm vent fluids and may provide attachment surfaces for microorganisms with chemoautotrophic metabolism.

Colonization of eggs of this species by epibiotic bacteria was recently described, and the lineages of *Gammaproteobacteria* and *Epsilonbacteraeota* found were the same as those retrieved within the cephalothorax of adults (Guri et al., 2012). This finding indicates that the bacteria-host relationship could start after the extrusion of the eggs by the female, during embryonic development (brooding phase).

Here we expand on Guri’s study by assessing variations in bacterial communities associated with broods of *R. exoculata* across different geographic locations, and at different stages through embryonic development. We also aimed to test the hypothesis of an establishment of the symbiotic relationship very soon after egg extrusion, by comparing bacterial communities developing on eggs with those developing on the pleopods holding the brood. Surfaces of both structures are co-localized on the female body and are thus assumed to be exposed to the same environmental conditions. If an opportunistic unspecific colonization (fouling) occurs on both structures, then we would expect similar bacterial assemblages. In such a case, a lack of specificity in egg-associated microorganisms would not support the hypothesis of the development of a symbiotic relationship at this stage. If egg surfaces trigger the attachment of specific microorganisms, then bacterial assemblages on pleopods would be expected to be significantly different from those developing on eggs. Such a pattern would be in favor of a selective mechanism, which could help establish symbiotic relationships very early in a shrimp’s life cycle.

In our study, bacterial assemblages that colonize the surfaces of eggs and of pleopods were analyzed in females holding broods at different stages of embryonic development collected from two different vent fields: Snake Pit (3460 m depth) and TAG (3630 m depth). The goals of this study were (i) to compare the bacterial assemblages found on the surface of the eggs and pleopods of brooding females; (ii) to assess variations in bacterial assemblages through embryonic development, and (iii) to compare the colonization of bacterial communities on eggs and pleopods from two different vent fields.

## Materials and Methods

### Sample Collection

**FIGURE 1:**
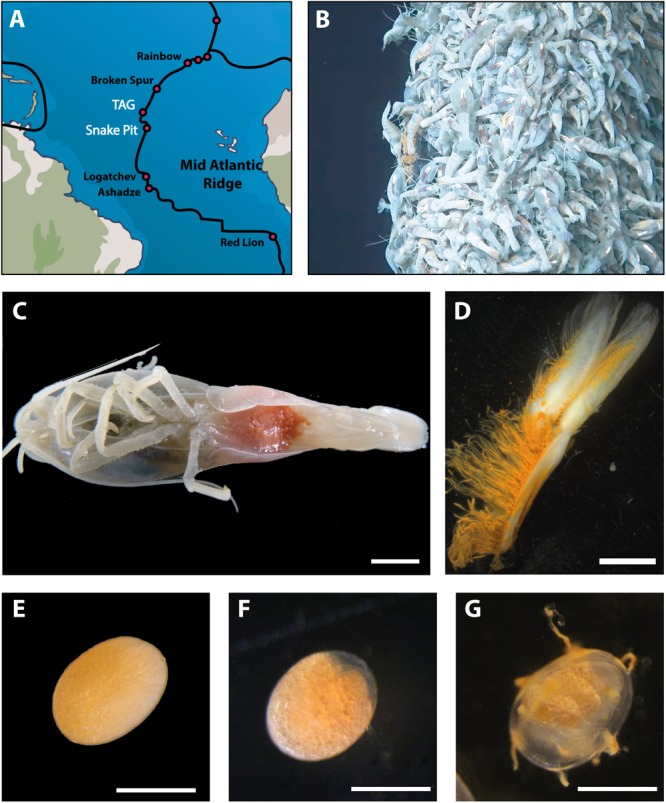
Sampling and dissection procedures. **(A)** Geographic location of hydrothermal vent sites where *Rimicaris exoculata* are present (black) and where brooding females were sampled (white). **(B)** Example of a shrimp aggregate where brooding females were collected at TAG vent field. **(C)** *Rimicaris exoculata* brooding female holding eggs between pleopods under the abdomen; scale bar = 5 mm. **(D)** First pleopod with modified setae dissected from a brooding female; scale bar = 2 mm. Single eggs **(E)** at early stage **(F)** middle stage **(G)**, and late stage of development; scale bars = 0.5 mm.

*Rimicaris exoculata* brooding females were collected at two vent fields on the MAR (Figure 1A), Snake Pit (23°22′N; 44°57′W, 3460 m depth) and TAG (26°08′N; 44°49.5′W, 3630 m depth), during the BICOSE (January 11 – February 10 2014) and BICOSE 2 cruises (January 26 2018 – March 10 2018). Specimens were collected in large aggregates inhabiting active emission habitats (Figure 1B), using a suction sampler manipulated by the Remote Operated Vehicle (ROV) Victor 6000 (BICOSE) or Human Operated Vehicle (HOV) Nautile (BICOSE 2).

Sampling at such depths implies that the collected organisms spend more than 1 h of ascent through the water column, before they are made available to scientists on board the oceanographic ship. Uncontrolled decompression during this ascent may considerably impair the physiological state of sampled fauna, leading to significant levels of mortality upon final recovery on board the ship. In order to improve the sampling conditions, pressurized recoveries of the specimens were performed using the PERISCOP^®^device: 18 such samplings were undertaken, 6 and 12 at the Snake Pit and TAG sites respectively (Supplementary Table S1). The specimens were collected inside a PVC sampling cell, which was connected to the slurp gun of the ROV or HOV. This sampling cell was further transferred to the isobaric chamber PERISCOP^®^before starting the ascent to the surface (Shillito et al., 2008). On board of the ship (R/V *Pourquoi pas?*) samples were rapidly decompressed in a controlled manner and processed for dissection and fixation. Survival rates were then close to 100%, as witnessed by the intense activity of the shrimp following the opening of the PERISCOP device.

Brooding females (Figure 1C) were selected from each site according to the developmental stage of the embryos in their brood. The eggs were classified into three developmental stages (Figure 1E–G):

– Early stage from one-cell embryos to fully segmented embryos (blastula);
– Middle stage includes embryos at the gastrulation (a clear area starts to be visible at one pole of the egg) to embryos showing early differentiation of larval structures;
– Late stage includes embryos with advanced development of larval structures and eyespots visible through the egg envelope.

For each female, the full brood was removed and two pleopods (Figure 1D) were dissected. A pleopod and a small number of eggs were fixed in 3% formalin for 3 h, rinsed with phosphate-buffered saline (PBS)/sterile seawater buffer (1:1) and stored in PBS/Alcohol at -20°C for FISH analyses (1:1) (Durand et al., 2010), while another pleopod and the rest of the brood were frozen at -80°C for DNA analyses.

In all, 40 *R. exoculata* samples were selected covering all combinations of body structure (brood vs. pleopod), developmental stage (early, middle, or late), and vent field (Snake Pit or TAG), each combination being replicated at least three times (Supplementary Table S1).

**FIGURE 2:**
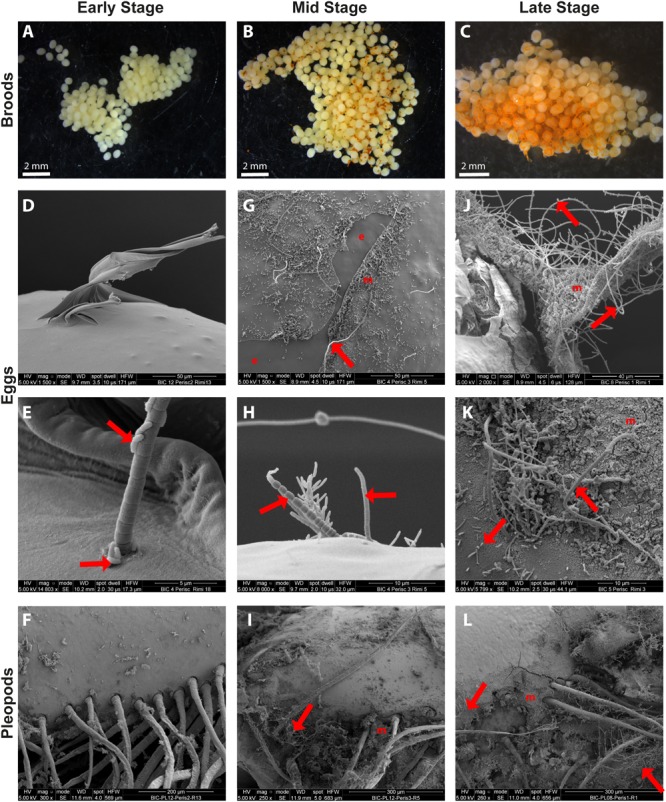
SEM observations of epibiotic bacteria on the surface of *R. exoculata* eggs and pleopods. *Rimicaris exoculata* egg broods observed under a stereomicroscope with eggs at **(A)** early stage, **(B)** mid stage, **(C)** and late stage. Individual eggs observed under a scanning electron microscope with eggs at **(D,E)** early stage, **(G,H)** mid stage **(J,K)** and late stage. Pleopods observed under a scanning electron microscope holding broods at **(F)** early stage, **(I)** mid stage, **(L)** and late stage. The crusts observed in the close-ups both on eggs **(K)** and pleopods **(L)** in SEM are deposits of ferric oxide, which correspond to the orange coloration observed under stereomicroscope on egg broods **(B,C)**. Red arrows are targeting some of the rod shaped and filamentous bacteria observed in these pictures. e, bare egg envelope areas where mucus layer have been removed and m, egg envelope areas covered by mineral crusts.

Other samples were also collected for complementary experiments (Supplementary Table S1) using scanning electron microscopy (SEM) and Fluorescent *In Situ* Hybridization (FISH). Samples were formalin-fixed as described above for the FISH analyses, or fixed in 2.5% glutaraldehyde in seawater, rinsed after 12 h of fixation and kept at 4°C in seawater for SEM analyses (Zbinden et al., 2004). For illustrative purpose (Figure 1, 2), images of egg broods, individual eggs and pleopods were taken under a stereomicroscope (Leica MS125, Leica Microsystems).

### DNA Extraction, Sequencing Method, and Sequence Processing

DNA from eggs and pleopods was extracted using a NucleoSpin^®^Soil Kit (Macherey-Nagel, Germany) following manufacturer’s instructions. The extracted DNA samples were then sent to the MR DNA laboratory (Molecular Research LP, Shallowater, TX, United States) for amplification and sequencing of prokaryotic diversity. This was performed on a 450 bp fragment of the 16S rRNA gene (16S) using Illumina’s MiSeq technology with the 2 × 300 bp chemistry. Briefly, the 16S V3-V4 variable region (Fadrosh et al., 2014) [341/785 primers, with barcode on the forward primer (Herlemann et al., 2011)] was amplified in a 28-cycle PCR using the HotStarTaq Plus Master Mix Kit (Qiagen, United States) under the following conditions: 94°C for 3 min, followed by 28 cycles of 94°C for 30 s, 53°C for 40 s and 72°C for 1 min, after which a final elongation step was performed at 72°C for 5 min. PCR products were then purified using calibrated Ampure XP beads and used to prepare a DNA library following the Illumina TruSeq DNA library preparation protocol. Blank DNA extraction controls were sequenced to verify there was no contamination, which was confirmed as these gave no amplification.

Prokaryotic 16S rRNA paired-end reads were merged using USEARCH (Edgar and Flyvbjerg, 2015) after q25 trimming of the ends. The resulting 16S reads were processed using FROGS, implemented on the Galaxy platform (Escudié et al., 2018), multifasta files were parsed, checked for quality, and trimmed by length (expected value of 455b ± 35b). Sequences were then clustered with SWARM (Mahé et al., 2014) using a distance threshold of three, chimeras were removed using the UCHIME algorithm (Edgar et al., 2011) implemented in VSEARCH (Rognes et al., 2016), and taxonomic affiliation was performed for each OTU by NCBI Blast+(Camacho et al., 2009) using the Silva 132 16S gene database (Quast et al., 2013). Additional filtering on abundance at the threshold of 0.005% (Bokulich et al., 2013) and on BLAST at 95% minimum coverage and 80% minimum identity was also performed to remove non-biologically relevant OTU sequences. Two OTUs affiliated to plant chloroplast and mitochondria (260 and 144 sequences of the total dataset, respectively) were manually removed from the dataset. The 16S rRNA data are available in the NCBI SRA repository (SubmissionID SUB4881289, BioProjectID PRJNA509116).

### Statistical Analysis

Alpha diversity within the 40 samples was estimated with OTU number and Inverse Simpson index (Simpson, 1949). Difference in the alpha diversity indexes among body structure and vent field categories were tested using Mann-Whitney tests and the difference between developmental stages, by Kruskal–Wallis followed by pairwise Wilcox tests; *p* < 0.05 was considered the threshold of significance for a difference between samples.

For further statistical analysis, the sequence dataset was normalized by cumulative sum scaling to minimize the effect of different sequence numbers obtained with each sample (Paulson et al., 2013). Beta diversity was analyzed by hierarchical clustering using the complete method based on Bray-Curtis dissimilarity matrices. The homogeneity between categories was tested with the betadisper function of the *Vegan* R package, and significant differences between categories were tested for by Permutational Analysis of Variance (PERMANOVA, 9999 permutations) with the adonis function of the same package (Oksanen et al., 2008). Multilevel comparisons for the developmental stage condition were also performed with the pairwise adonis function (Arbizu, 2017).

Taxonomic composition and diversity results were visualized using the R package *Phyloseq v.1.14.0* (McMurdie and Holmes, 2013). The linear discriminant analysis (LDA) effect size (LefSE) method (Segata et al., 2011) was used to characterize and highlight microorganisms specific to each of the different conditions. Results were shown both with a plot of LDA score for each bacterial group and with a cladogram, each concentric circle representing a taxonomic level, the innermost being the phylum and the outermost the genus.

### Scanning Electron Microscopy

Eggs and pleopods were dehydrated with an ethanol series (30, 50, 70, 95, and 100% ethanol) and then for 5 h in a critical point dryer CPD 020 (Balzers Union, Balzers, Liechtenstein). Finally, samples were gold-coated with an SCD 040 (Balzers Union). Observations and imaging were performed using a Quanta 200 microscope (FEI-Thermo Fisher, Hillsboro, OR, United States). The chemical composition of the mineral crust present on egg and pleopod surfaces was also analyzed with an X-ray Energy Dispersive Spectrometer (EDX) using an X-Max 80 (Oxford Instruments, Oxford, United Kingdom) and displayed with AZtec software (Oxford Instruments, Oxford, United Kingdom).

### Fluorescent *in situ* Hybridization

Eggs were dehydrated with a PBS-Ethanol series (from 50:50 to pure ethanol with a 10% increase for each bath, 10 min each) and progressively transferred to LR-white resin in an Ethanol-Resin series (2:1, 1:1, and 1:2 Ethanol/Resin ratio; 30 min each). After three baths of 30 min in pure LR-white resin to remove any trace of ethanol, samples were placed in gelatin capsules filled with LR-white and left for 48h for further impregnation at room temperature. The LR-white resin was polymerized in the gelatin capsules at 50°C for 24 h. Before sectioning, the gelatin was removed with hot water and the LR-white blocks were cut into 2-μm sections in series through the entire egg or half of it using an ultramicrotome Ultracut UCT (Leica).

Sections were mounted on glass slides, and hybridized in a reaction mixture containing 0.5 mM of each probe in a 30% formamide hybridization buffer [0.9M NaCl, 0.02M Tris-HCl, 0.01% sodium dodecyl sulfate (SDS), and 30% deionized formamide] for 6 h at 46°C. Different concentrations of formamide (10, 20, 30, 40, and 50%) were initially tested, but 30% formamide always gave the best results. Hybridization temperature was chosen according to Guri et al. (2012). Sections were washed at 48°C for 30 min in a washing buffer (0.102M NaCl, 0.02M Tris-HCl, 0.005M EDTA, 0.01% SDS) and rinsed briefly with water. Sections were covered with Slow Fades Gold antifade reagent containing 40-6-diamidino-2-phenylindole (DAPI) (Invitrogen), and a cover slip. The probes used in this study (Eurofins) were Eub338, targeting most of the *Eubacteria* (Amann et al., 1990); GAM42a, targeting the *Gammaproteobacteria* (Manz et al., 1992); Epsy914, targeting *Epsilonbacteraeota* (Loy et al., 2003); and Zeta123, targeting *Zetaproteobacteria* (Kato et al., 2009); and labeled with Cyanine 3 or Cyanine 5 (Supplementary Table S2).

Observations were made on a Zeiss AxioImager.Z2 microscope equipped with Apotome, an AxioCam, and a Colibri LED light source with three light-emitting diodes (UV-emitting LED, 365 ± 4.5 nm for DAPI; green emitting LED, 550 ± 14 nm for Cyanine3; red-emitting LED, 590 ± 17.5 nm for Cy5) (Carl Zeiss MicroImaging GmbH, Göttingen, Germany). Micrographs were analyzed using Zen (Zeiss) software.

## Results

### Electron Microscopy Analysis

Observed under a light microscope, early stage broods were almost devoid of mineral deposits, except for some thin and scattered mineral deposits found on some eggs (Figure 2A). Conversely, middle stage broods had small patches of mineral deposits (Figure 2B). Mineral deposits were even greater on late stage broods, some of which were almost half covered with a thick mineral crust (Figure 2C). Eggs in these late broods showed high variation in mineral coverage, with more minerals covering eggs of the external part of the brood (Figure 2C).

As shown in the SEM images, the surface of early stage eggs was colonized by only a few scattered bacteria (Figure 2D). The morphotypes identified at this stage were small isolated rod-shaped bacteria, mostly attached laterally, and 1-μm thick filaments with a proximal attachment to the eggs, measuring 10 s to 100 s μm (Figure 2E). The surface of pleopods holding these early stage broods was also colonized by a few rod-shaped and filamentous bacteria (Figure 2F).

At the middle stage, the broods showed different degrees of bacterial colonization, usually with patches of bacteria over the eggs and on the connective mucus. In general, the bacterial coverage was denser than on early stage broods, with more bacterial filaments, including thin (<1 μm) and thick (>1 μm) filaments and erect rod-shaped bacteria (Figure 2G,H). In sections where the mucus coat was broken, allowing observation of the egg envelope itself, mostly, no bacteria directly attached to the egg envelope were observed (Figure 2G). In addition, in some areas, the bacteria were found with crusts of mineral deposits of different thickness (up to 5 μm). Spectrophotometric analysis of this mineral crust showed that the deposits were mainly composed of ferric oxides for both eggs and pleopods (Supplementary Figures S1D,E). Similar bacterial colonization was also observed on pleopod surfaces (Figure 2I).

On broods in the late stage of embryonic development, bacteria covered the eggs almost entirely. The morphotypes identified were the same as those found on middle stage eggs (Figure 2J,K). However, the distribution was more uniform over the eggs and connective mucus. Similarly, there were more mineral deposits, which tended to form thick crusts (5–7 μm thick), covering even the connective mucus. In some spots with mineral deposits, it seemed that the crust covered a part of the bacterial biofilm, but there were still bacterial cells visible over the crust. The surface of pleopods holding late stage broods were also covered by a thick crust of mineral deposits and colonized by several thick and thin filaments similar to those found on eggs (Figure 2L). Bacterial colonization on the eggs or pleopods during embryonic development showed similar patterns in the TAG and Snake Pit vent fields. As for middle stages, spectrophotometric analysis of the thick mineral crust confirmed presence of ferric oxides on both late stage egg and pleopod surfaces (Supplementary Figures S1A–C).

### Microbial Diversity Analysis

Our metabarcoding of the bacterial communities associated with eggs or pleopods of *R. exoculata* generated a total of 3,361,475 sequences. After sequence filtering, there remained a total of 2,269,630 partial 16S RNA (V3-V4 regions) sequences clustered in 228 OTUs to assess bacterial diversity. Most sequences were identified on both body structures, with only one OTU and one bacterial order of Mollicutes specific to eggs, namely the *Entomoplasmatales* (Supplementary Figure S2). In the same way, two OTUs were specific to Snake Pit and one order of Mollicutes specific to TAG, namely the *Mycoplasmatales*, the rest being common to both vent fields (Supplementary Figure S2). All OTUs except one, absent from late stage samples, were present at every developmental stage (Supplementary Figure S2).

None of our samples had difference in average bacterial OTU number according to surface type (egg vs. pleopod), vent site or developmental stage (Supplementary Figure S3). However, Inverse Simpson values indicated a greater mean evenness in egg samples compared with pleopod samples (*p* = 0.001041; Supplementary Figure S3A). Pleopods were dominated by fewer OTUs whereas eggs had more even microbial communities in comparison. Interestingly, a shift could be observed through the developmental stages with a significantly higher evenness for late stage pleopods compared with middle and early stages (*p* = 0.003; Supplementary Figure S3E). Similar variation was observed for eggs with a slight increase in average Inverse Simpson values for late stages compared to early and middle stages, although supported by a marginal *p*-value (*p* = 0.052; Supplementary Figure S3D). No significant differences were found between TAG and Snake Pit samples for eggs or pleopods (Supplementary Figures S3B,C).

Regardless of the vent site, egg, and pleopod communities were significantly different from each other (PERMANOVA *R*^2^ = 0.233, *p* < 0.001, Supplementary Table S3). Body surface (egg vs. pleopod) was also the main factor structuring our dataset, followed by vent site effect (*R*^2^ = 0.195) and developmental stage effect (*R*^2^ = 0.148, Supplementary Table S3). Taken separately, both egg and pleopod surfaces, showed a strong vent site effect on bacterial community composition, with significant differences between TAG and Snake Pit assemblages (PERMANOVA *R*^2^ 0.291 and 0.316 *p* < 0.001 for both eggs and pleopods).

In addition, significant variations in bacterial communities were detected through the egg development stages (PERMANOVA *R*^2^ 0.263 and 0.308, *p* = 0.004 for both eggs and pleopods). These variations were mainly observed between early stages and late stages for both surfaces (Pairwise PERMANOVA *R*^2^ 0.299 and 0.379, *p* = 0.013 and 0.009 for eggs and pleopods, respectively). Indeed, no significant differences were found between middle and late stage for either surface, or between early and middle stages for pleopods. Only slightly significant variations were found between early stages and middle stages for eggs (Pairwise PERMANOVA, Supplementary Table S3).

**FIGURE 3:**
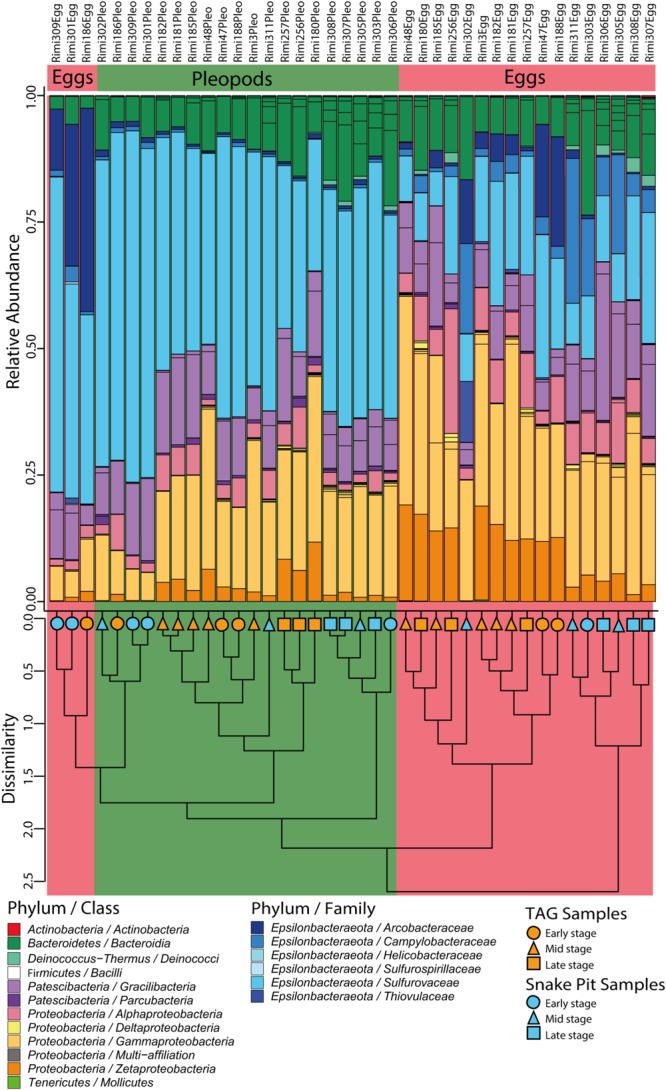
Relative abundances of 16S rRNA gene sequence reads from egg and pleopod samples according to their classification (Silva 132 database). Groups are at the family level for the *Epsilonbacteraeota* phylum and at the class level for other phyla. The cluster dendrogram depicts the average linkage hierarchical clustering based on a Bray-Curtis dissimilarity matrix of community compositions resolved down to OTU level.

These results were further supported by hierarchical clustering, which separated most Snake Pit eggs from all other samples and then most TAG eggs from the rest, most of which were pleopod samples (Figure 3). Only some early stage egg samples (from both sites) clustered together with early stage pleopods in a separate cluster (Figure 3). In any case, body surface samples (i.e., eggs vs. pleopods) or vent site origin, were more closely related to each other than were samples originating from the same brooding female.

### Microbial Taxonomic Comparison

Based on rDNA 16S sequence affiliation according to the Silva 132 database, nine phyla could be identified in our samples, with just four phyla accounting for more than 99% of the sequences obtained (Figure 3). The most abundant taxa were *Epsilonbacteraeota*, representing in 39% of the total dataset in relative abundance, followed by *Proteobacteria* (36%), *Patescibacteria* (13%), and *Bacteroidetes* (11%). Among *Proteobacteria*, three major classes were present with a clear dominance of *Gammaproteobacteria* (63.7%), followed by *Zetaproteobacteria* and *Alphaproteobacteria* (17.7% each). Sequences affiliated to *Rothia* sp. from the *Actinobacteria* phylum (0.007%), *Streptococcus* sp., and *Enterococcus* sp. from the *Firmicutes* phylum (0.013%) were only present in a few samples and most likely correspond to potential contaminants. In the same way, two other phyla, *Tenericutes* (0.025%) and *Deinococcus–Thermus* (0.32%) were present in only a few samples.

**FIGURE 4:**
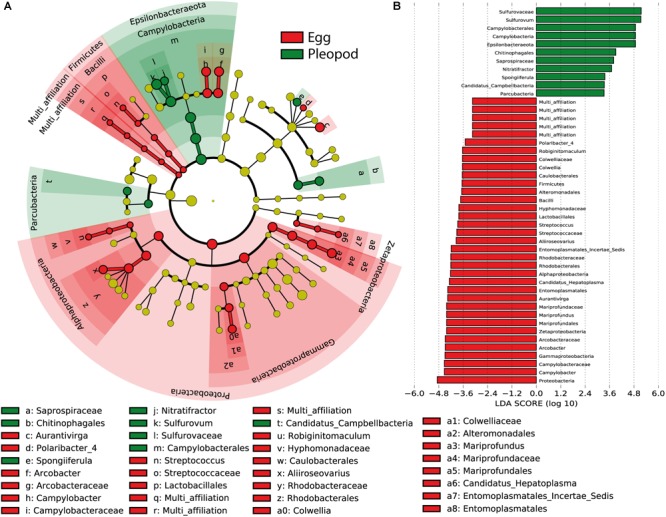
Most differentially abundant bacterial taxa between eggs (red) and pleopods (green). **(A)** Cladogram representing taxonomic distribution of the differentially abundant bacterial taxa. The dots represent the specific bacterial taxa, with colors corresponding to the surface (eggs or pleopods) where these are relatively more abundant, yellow-greenish color denoting taxa with no significant difference in relative abundance between eggs and pleopods. The size of dots is proportional to the relative abundance. **(B)** Ranked LDA scores of the differentially abundant bacterial taxa, with taxa with highest relative abundance in pleopods in green, and taxa with highest relative abundance in eggs in red.

One of the major differences between bacterial communities on eggs and pleopods was the relative proportion of the different *Epsilonbacteraeota* families. As shown by LDA analysis (Figure 4 and Supplementary Figure S4), *Epsilonbacteraeota* were significantly more abundant in pleopod than in egg communities (49 and 36%, respectively). Additionally, among this phylum, members of the *Sulfurovaceae* family were much more abundant in pleopod (97.8% of the relative abundance of all *Epsilonbacteraeota*) than in egg communities (57.5% of all *Epsilonbacteraeota*). Conversely, members of the *Arcobacteraceae* and *Campylobacteraceae* families were much more abundant in egg (22.1 and 18% of all *Epsilonbacteraeota*, respectively) than in pleopod communities (less than 1% for both). The relative abundance of *Zetaproteobacteria* (8% in eggs compared with 3% in pleopods) also contributed to the difference between egg and pleopod communities (Figure 4). Despite their relatively high abundance, *Gammaproteobacteria* show similar relative abundance with no significant differences in both eggs and pleopod communities (24 and 19%, respectively). Only one *Gammaproteobacteria* genus, *Colwellia*, was shown by the LDA analysis to be significantly more abundant in eggs. This result was the effect of a single brooding female – Rimi185 – on whose eggs the *Colwellia* family accounted for 50% of the total *Gammaproteobacteria* class. In other samples, including the pleopods of Rimi185, *Colwellia* were always present in low abundance and the *Gammaproteobacteria* class was mainly dominated by *Thiotricaceae*. Interestingly, as illustrated by Venn diagrams (Supplementary Figure S2), our LDA analysis confirms Mollicutes from the *Entomoplasmatales* order as an egg-specific group present only in some of our egg samples.

**FIGURE 5:**
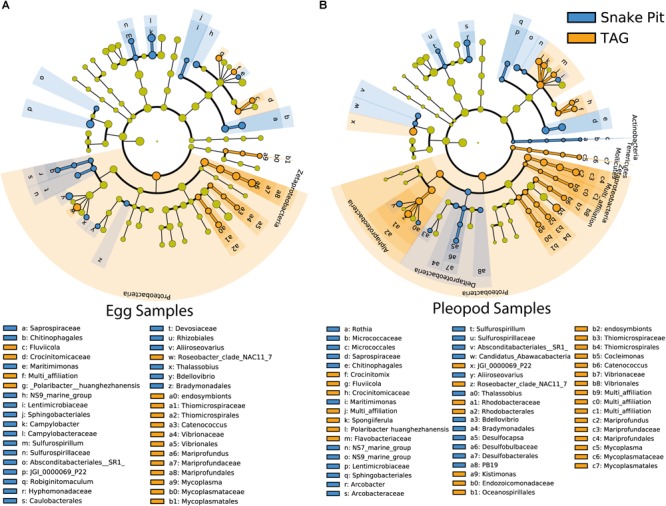
Most differentially abundant taxa of egg communities **(A)** and pleopod communities **(B)** between TAG (orange) and Snake Pit (blue) vent fields. The dots represent the specific bacterial taxa, with colors corresponding to the vent field where these are relatively more abundant, yellow-greenish color denoting taxa with no significant difference in relative abundance between vent fields. The size of dots is proportional to the relative abundance.

Regarding vent site influence, LDA analysis (Figure 5 and Supplementary Figure S5) revealed that *Zetaproteobacteria* (13 and 3% for TAG and Snake Pit, respectively) *Campylobacteraceae* (2 and 11% for TAG and Snake Pit, respectively), and the JGI_00000069_P22 order of the *Gracilibacteria* family (6 and 13% for TAG and Snake Pit, respectively), were the main lineages responsible for the difference between the two vent fields in egg communities (Figure 5A). By contrast, *Alphaproteobacteria*, mainly from the *Rhodobacterales* order (8 and 3% for TAG and Snake Pit, respectively), *Zetaproteobacteria* (8 and 2% for TAG and Snake Pit, respectively), and the *Bacteroidetes* family of *Saprospiraceae* (9 and 6% for TAG and Snake Pit, respectively) were the main lineages responsible for the difference between the two vent fields in pleopod communities (Figure 5B).

**FIGURE 6:**
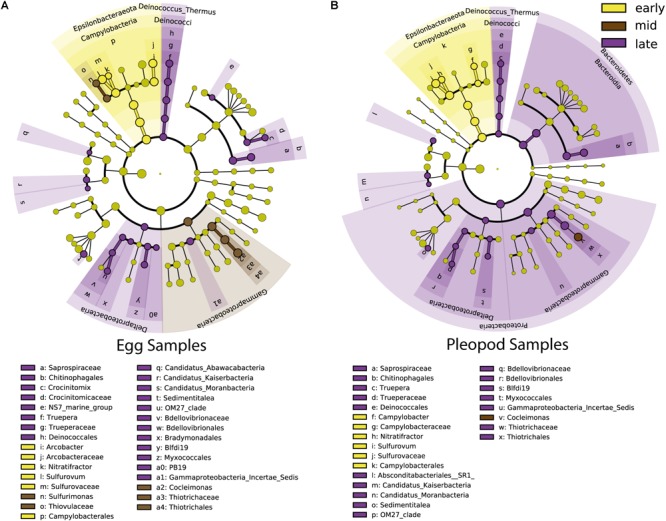
Most differentially abundant taxa of egg communities **(A)** and pleopod communities **(B)** between early stages (yellow), middle stages (brown) and late stages (purple). The dots represent the specific bacterial taxa, with colors corresponding to the stage where these are relatively more abundant, yellow-greenish color denoting taxa with no significant difference in relative abundance between stages. The size of dots is proportional to the relative abundance

During egg development, an important shift between *Epsilonbacteraeota* and *Gammaproteobacteria* was observed, *Epsilonbacteraeota* being more abundant at early stages (from 58 to 23% for eggs and from 56 to 33% for pleopods) and *Gammaproteobacteria*, more abundant at late stages (from 15 to 26% for eggs and from 15 to 24% for pleopods) (Figure 6A,B and Supplementary Figure S6). Increased abundance at late stages for *Deinoccocus–Thermus* (from less than 0.1 to 2% for both eggs and pleopods), *Bacteroidetes* family *Saprospiraceae* (from less than 0.1–4% for eggs; 6% for pleopods), and *Deltaproteobacteria* (from less than 0.1–1% for both eggs and pleopods) lineages were also highlighted by LDA analysis (Figure 6A,B and Supplementary Figure S6).

### Fluorescent *in situ* Hybridization Observations

**FIGURE 7:**
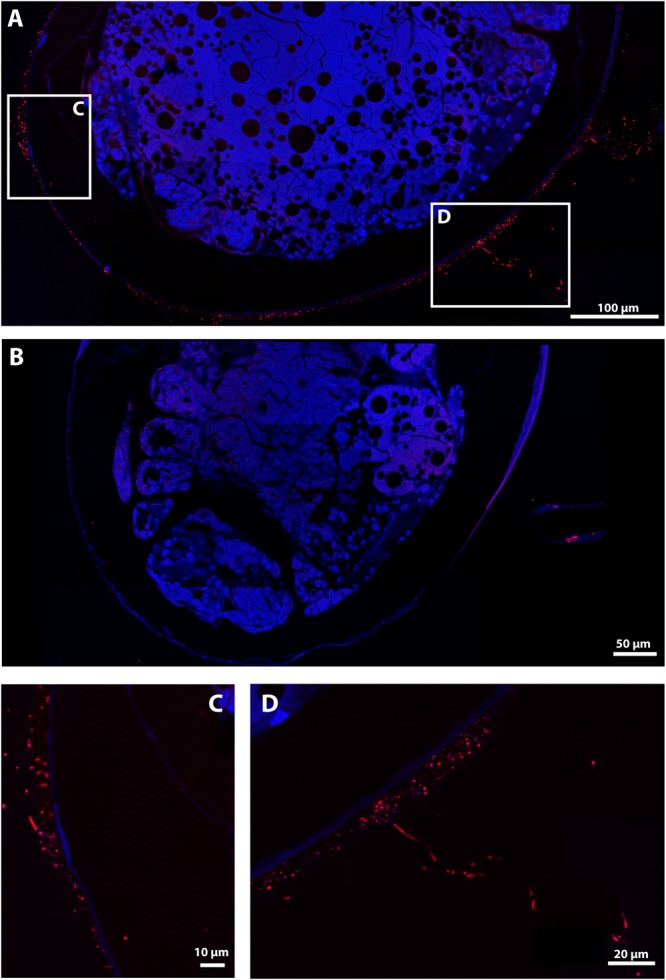
FISH Observations of *Rimicaris exoculata* late stage eggs with universal bacteria probes. Observations were performed on semi-thin sections (2 μm) stained with DAPI (blue) and hybridized with Eub338-Cy5. **(A,C,D)** Egg from the external part of the brood with abundant mineral deposits, **(B)** Egg from the internal part of the brood without mineral deposits, Colors = Red (*Eubacteria*).

On the hybridized sections, a positive signal with the *Eubacteria* probe was obtained on the envelope of all late stage eggs covered with mineral deposits, showing thin filaments and rod-shaped structures (Figure 7A). By contrast, no positive signal was obtained with the *Eubacteria* probe on most hybridized sections of early stage eggs (Supplementary Figure S7), confirming SEM observations that bacterial assemblages are hardly present at all at this stage.

At the late stage, however, eggs from the same brood showed high variation in terms of bacterial coverage, showing some eggs with abundant mineral deposits, and others without any mineral deposit on their envelope. The first type had a large number of filamentous bacteria and rod-shaped bacteria (Figure 7A,C,D), whereas those without mineral deposits had less bacteria, more similar to the early stage, with only a few rod-shaped erected bacteria revealed by a weak Eub338 signal (Figure 7B). Differences in the abundance of mineral deposits on the eggs were associated with egg position in the brood. The egg with many mineral deposits being the most external and the most exposed to the hydrothermal fluids.

**FIGURE 8:**
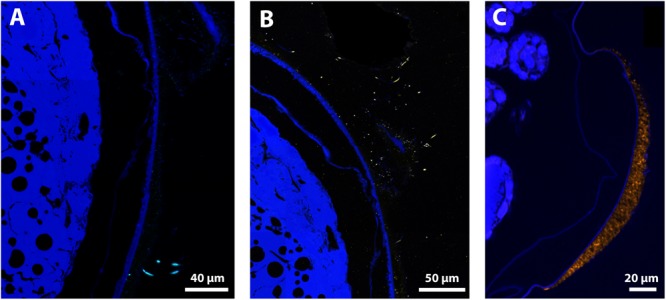
FISH Observations of *Rimicaris exoculata* late stage eggs with specific probes. Observations were performed on semi-thin sections (2 μm) stained with DAPI (blue) with eggs from the external part of the brood with abundant mineral deposits, hybridized **(A)** with Epsi914-Cy5, **(B)** with Gam42a-Cy5, and **(C)** with Zeta123-Cy3. Colors = Light Blue (*Epsilonbacteraeota*), Yellow (*Gammaproteobacteria*), and Orange (*Zetaproteobacteria*).

Positive signals were obtained with the *Epsilonbacteraeota* probe on the thick filaments and with the *Gammaproteobacteria* probe on thin filaments for late stage eggs, confirming their status as active bacteria on egg surfaces (Figure 8A,B). Positive signal with the *Zetaproteobacteria* probe was also obtained for bacteria entangled within the mineral crust for late stage eggs (Figure 8C). Moreover, the *Gammaproteobacteria* signal matched with most of the thin filamentous bacteria and the small-rod bacteria observed with DAPI, corroborating their high abundance at these stages (Supplementary Figure S7). This was further supported by co-hybridization with *Gammaproteobacteria* and *Eubacteria* probes, which showed that a *Gammaproteobacteria* signal co-localized with most of the *Eubacteria* signals (Supplementary Figure S7). Considering the low bacterial coverage at the early stage, no consistent signal could be observed with specific probes (*Gammaproteobacteria* or *Epsilonbacteraeota*). For each of these groups, no signal with specific probes was reported inside the eggs or elsewhere than on the surface of egg envelopes (Figure 8 and Supplementary Figure S7).

## Discussion

When the bacterial association with eggs of *R. exoculata* was first described, in Guri et al. (2012), lineages of *Gammaproteobacteria, Epsilonbacteraeota, Alphaproteobacteria*, and *Bacteroidetes* similar to those retrieved within the cephalothorax of adults were reported. In our present study, in addition to the previous ones, symbiont-related lineages of *Zetaproteobacteria* or *Entomoplasmatales* previously reported in *R. exoculata*, cephalothorax or gut were also retrieved (Zbinden and Cambon-Bonavita, 2003; Jan et al., 2014). For three of these, namely the *Gammaproteobacteria, Zetaproteobacteria*, and *Epsilonbacteraeota*, localization on the egg surface close to mineral deposits was confirmed here by FISH (Figure 8). Our results also highlight significant differences between the bacterial assemblages found on eggs and on pleopods, as well as a significant effect of the mother’s vent of origin and the developmental stage of the brood.

### Variation in Bacterial Assemblages Through *Rimicaris exoculata* Embryonic Development

At the early stage, egg and pleopod surfaces have low bacterial coverage, with only a few rod-shaped and filamentous bacteria shown by SEM images, and no positive signal with FISH bacteria-specific probes. These small assemblages already include almost every bacteria found throughout embryonic development. Nonetheless, most assemblages are mainly dominated by *Epsilonbacteraeota*, other phyla being present only in low relative abundance at this developmental stage. High abundance of this group on newly available surfaces, i.e., freshly laid eggs and recently molted pleopods, is in good agreement with previous studies reporting *Epsilonbacteraeota* as pioneer colonizers on several types of substratum available at vents after just a few days of colonization (López-García et al., 2003; Alain et al., 2004; Szafranski et al., 2015a).

As embryonic development progresses, bacterial coverage and mineral deposits both appear to follow a gradual progression on eggs and pleopods, clearly reminiscent of what is observed inside the cephalothorax after molting (Zbinden et al., 2004; Corbari et al., 2008b). This increase of bacterial coverage at later stages is accompanied by important changes in community composition. For both types of body surface, higher Inverse Simpson index suggests a more even distribution and a higher diversity in bacterial assemblages at the late stage than at the early or middle stages. Although *Epsilonbacteraeota* may be more abundant in late stage communities than in small early stage assemblages, relative abundance clearly decreases in favor of other groups. Among these, *Gammaproteobacteria, Deltaproteobacteria*, and *Deinococcus–Thermus* were significantly more abundant on late stage surfaces, resembling the most complex communities found on long-term colonized surfaces of the MAR (Szafranski et al., 2015a).

Molting of caridean brooding females can be postponed in some cases until the end of embryonic development and hatching of the larvae, but not in every species and never for more than a few days (Hess, 1941; Pandian et al., 1982; Bauer and Abdalla, 2000; Raviv et al., 2008). In *R. exoculata*, the time period between two successive molts in non-reproductive individuals has been estimated to be rather short, around 10 days (Corbari et al., 2008b). Egg hatching in the Alvinocaridid shrimp *Shinkaicaris leurokolos* incubated experimentally at temperatures representative of the adult habitat (around 20°C) occurred within a month (Watanabe et al., 2016), and the brooding period in *R. exoculata* likely has similar duration, thus extending their usual intermolt period to a few weeks at most. Therefore, it is interesting to observe that colonization patterns in our samples are different from those observed in studies with a similar exposure period – approximately 2 weeks – both for experimental colonization devices and for other cuticular surfaces of the same species (Guri et al., 2012; Szafranski et al., 2015a; Zbinden et al., 2018). Rather than being dominated by *Epsilonbacteraeota*, our late stage samples, especially the eggs, show bacterial assemblages with higher evenness that are closer to those of long-term colonized surfaces deployed for about a year on MAR vent sites (Szafranski et al., 2015a).

Although a full understanding of all the drivers influencing colonization over egg development phases is not provided here, we can propose an explanation of colonization by some bacterial groups, arising from a combination of environmental influences and female behavior. *Epsilonbacteraeota* and thiotrophic *Gammaproteobacteria* are often described as sulfur oxidizers thriving in close but differing environmental niches due to their metabolic capacities (Petersen et al., 2010; Meier et al., 2017). Members of the first group, which predominantly use the rTCA cycle, are described as anaerobic or microaerophilic sulfur oxidizers, whereas members of the second group use the CBB cycle and are able to grow at higher O_2_ concentration (Nakagawa and Takai, 2008). Additionally, many caridean shrimps and other decapods actively ventilate their egg mass by moving the pleopods frequently and quickly, with the frequency of this ventilation behavior increasing as the embryos mature (Fernández et al., 2002; Reinsel et al., 2014). A faster ventilation on late stage broods of *R. exoculata* could therefore result in a boost of oxygen supply to both egg and pleopod surfaces, promoting the growth of *Gammaproteobacteria* at the expense of *Epsilonbacteraeota*. *Zetaproteobacteria* are iron oxidizers that also use the CBB cycle (Field et al., 2014; Jan et al., 2014). However, they do not show significant abundance increase in response to increased mother ventilation with brood development. The increased oxygen supply could favor abiotic iron oxidation rather than bacterial metabolism, thus counterbalancing *Zetaproteobacteria* growth which remains approximately stable during egg development.

With microscopy, finer-scale observations revealed another level of variation when different eggs from the same brood were compared. As highlighted by FISH results (Figure 7), late stage eggs from the outer part of the brood have more mineral deposits and are colonized by more bacteria than late stage eggs from the inner part of the brood, for which bacterial coverage on the envelope is almost non-existent. Arguably, bacterial coverage on the egg envelope seems to be driven by both the position of the egg within the brood and the egg developmental stage, which ultimately reflects the degree of exposure of an egg to vent fluids. Therefore, not all eggs of *R. exoculata* are found to have a close association with bacterial assemblages on their envelope. Bacterial association at this stage of the animal life cycle should therefore perhaps be considered at the scale of the mother for the entire brood rather than at an individual scale for each embryo. The way in which this relationship evolves after hatching of *Rimicaris* larvae cannot be clarified with currently available data.

### Bacterial Assemblages of *Rimicaris exoculata* Eggs Are Influenced by Vent Fluid Chemistry

A sharp contrast between the bacterial communities in TAG and Snake Pit vent sites also underlines the importance of environmental influences on these assemblages. Geographical isolation has been proposed in some studies to explain the existence of distinct bacterial communities between different vent sites (Huber et al., 2010; Akerman et al., 2013). It is more likely, however, that variations in vent fluid chemistry could explain the differences between TAG and Snake Pit assemblages. Indeed, with the exception of *Mycoplasmatales*, an order found only in TAG samples, or some *Cand. Campbellbacteria* OTUs present only at Snake Pit (Supplementary Figure S2), every other OTU was shared between communities from both vent sites, invalidating the hypothesis of a geographical exclusion. In addition the absence of these two groups in either TAG or Snake Pit samples could also be related to an insufficient sequencing depth since they were present in low abundance in our dataset.

A striking case supporting the major influence of vent fluid chemistry is that of *Zetaproteobacteria*, a group of iron-oxidizing bacteria that were significantly more abundant in TAG than in Snake Pit samples, both on egg and pleopod surfaces. Vent fluids from TAG are described as having much higher concentrations of Fe, Cu, and Mn than Snake Pit, which are richer in Cd and Pb (Jean-Baptiste et al., 1991; Tivey et al., 1995; Fouquet et al., 2010). Thus, a higher relative abundance of *Zetaproteobacteria* on both surfaces at TAG could result from a higher concentration of the energy source that fuels their metabolism. This is mirrored by observations made by Guri et al. (2012) on egg broods from Logatchev. In this vent site, fluids are enriched in CH_4_ compared with TAG and Snake Pit, where CH_4_ concentrations are very low (Charlou et al., 2002; Fouquet et al., 2010). Whereas no sequences of methanotrophic *Gammaproteobacteria* were found in our samples, a high number of sequences of this particular group were retrieved from clone libraries from Logatchev egg broods. Similar variations according to vent fluid composition were also described for symbiont communities within the cephalothorax of adult *R. exoculata* (Guri et al., 2012; Jan et al., 2014) and are not uncommon even in endosymbiotic communities of other vent species. Bathymodiolin species of the MAR, for example, show high flexibility in their relationship with two *Gammaproteobacteria* partners with distinct metabolic capacities. Variations in relative abundance of methane or sulfur-oxidizing symbionts were observed according to the fluid chemistry of their vent site of origin, but also according to the availability of these chemical substrates when mussels were incubated experimentally in pressure vessels (Duperron et al., 2006; Szafranski et al., 2015b). Such cases emphasize the important effect of vent fluid chemistry on the diversity observed in bacterial assemblages from specific vent sites.

Positive interactions between two or more of the bacterial partners could also explain the difference in relative abundance between TAG and Snake Pit communities in some lineages that are apparently not favored by local geochemistry. In such cases, if one of the partners is found to be more abundant in response to favorable vent fluid composition, the other partner could also be more abundant but not as a direct impact of the vent fluid chemistry. Such an interaction was previously observed between *Epsilonbacteraeota* and *Bacteroidetes* (Stokke et al., 2015), and could explain the higher abundance of some *Bacteroidetes* lineages in Snake Pit samples compared with those from TAG. Additional samples of brooding females and comparison of egg and pleopod assemblages from other vent sites with a greater difference in fluid composition, such as the ultramafic vent sites of Rainbow or Logatchev, would provide more evidence on the relative influence of vent fluid and body surface factors on the microbial community composition.

### Is There a Specific Bacterial Assemblage Associated With *Rimicaris exoculata* Egg Broods?

Despite of the importance of vent fluid chemistry, bacterial assemblages developing on eggs and pleopods were significantly different whatever their vent site of origin or developmental stage. Surprisingly, although they have been reported as the dominant symbionts within the *R. exoculata* cephalothorax in many studies (Petersen et al., 2010; Hügler et al., 2011; Guri et al., 2012), establishment of *Epsilonbacteraeota* from the *Sulfurovaceae* family was not as favored on egg envelopes as it was on pleopods. On the other hand, other families from this phylum, namely the *Arcobacteraceae* and the *Campylobacteraceae* had higher abundances on egg envelopes. Likewise, *Thiotricaceae* from the *Gammaproteobacteria* phylum, another important group retrieved within the cephalothorax, was not specifically associated with egg broods, being as abundant on their surfaces as on pleopods.

By contrast and despite their higher abundance in TAG bacterial assemblages, *Zetaproteobacteria* were always relatively more abundant on egg broods than on pleopod surfaces. *Zetaproteobacteria* have already been reported in close association with *R. exoculata* but never in such high relative abundance (Jan et al., 2014). They are often described as ecosystem engineers due to their capacity to shape the local environment by producing iron oxyhydroxide sheaths and stalks (Chan et al., 2011; Fleming et al., 2013; Scott et al., 2015). These structures provide architecture for the mats, which can alter local geochemistry, enhancing microbial diversity. For example, *Zetaproteobacteria*-dominated mats showed significantly higher diversity, both in terms of OTU numbers and evenness, than other bacterial mats (Hager et al., 2017). A significantly higher evenness of egg surfaces compared to pleopods could also result from the higher abundance of *Zetaproteobacteria* observed in our egg brood samples. All these results indicated a clear difference between egg and pleopod bacterial communities, supporting the existence of a specific brood microbial assemblage that differs from a simple opportunistic colonization on the egg surface but also from the symbiotic community within the adult cephalothorax.

This divergence between egg and pleopod microbial communities could be linked to distinct physical properties such as surface energy, hydrophobicity, and roughness between the hard exoskeleton cuticle of pleopods and the soft envelope of *R. exoculata* eggs. These factors have indeed been observed in laboratory experiments as playing an important role in determining which microorganisms could successfully attach to a specific substrate (Cooksey and Wigglesworth-Cooksey, 1995). Nonetheless, several studies with colonization devices conducted on deep sea ecosystems, noted little impact of substrate type on the microbial community composition compared with other environmental factors (Bellou et al., 2012; Lee et al., 2014; Szafranski et al., 2015a). Other characteristics of *R. exoculata* eggs could therefore offer another explanation for this divergence between bacterial assemblages of different host surfaces. As stated in a previous study (Guri et al., 2012), our SEM observations confirmed the presence of a mucus-like material surrounding the eggs at every developmental stage (Figure 2G). This mucus coat seems to allow bacteria to attach since in areas where this mucus coat was lacerated bacteria were absent. Several studies have shown the importance of mucus for inter-partner recognition and for microorganism selection by many symbiotic metazoan hosts (Nyholm and McFall-Ngai, 2004; Nussbaumer et al., 2006; Bright and Bulgheresi, 2010). This selection usually implies tight chemical communication involving many molecules produced by the host, such as surface sugars, sugar-binding proteins or antimicrobial peptides (AMPs), all of which are important components of host mucus matrices (Bright and Bulgheresi, 2010; Rosa and Barracco, 2010). Such chemical communication molecules, the *luxS* and *luxR* genes, were already reported for *Epsilonbacteraeota* and *Gammaproteobacteria* symbionts of *R. exoculata* and are likely to be involved in the regulation of the symbiont population (Le Bloa et al., 2017). We hypothesize that similar mechanisms exist in *R. exoculata* during embryonic development and that a differential and regulated positive selection between host surfaces could explain differences observed between egg and pleopod bacterial communities. Future investigations on potential mechanisms, such as production and localization of potential AMPs within the brood or other aspects of shrimp maternal immunity are needed to understand these processes of selection more clearly.

### Perspectives on Roles and Acquisition of Bacteria Through the *Rimicaris* Life Cycle: What Gets Colonized First, the Egg or the Shrimp?

Our results provide first insights about bacterial colonization on different body surfaces of *R. exoculata*, which might be a host-controlled process. If such a selection exists, there must be an associated cost for the animal, counterbalanced by a potential role enhancing its fitness. The production of a cocktail of molecules by the mother or developing eggs to select specific bacteria could be one of these processes, and maternal behavioral responses, such as increased ventilation along egg development could be another. For adult shrimps, a nutritional role is now established for the symbiosis within the cephalothorax, which harbors bacterial lineages similar to those found in our work (Ponsard et al., 2013). However, absence of direct association for some eggs within the brood – those of the inner area – and high abundance of lipid reserves maintained even after hatching in *Rimicaris* larvae (Hernández-Ávila et al., 2015) suggest that a similar role is quite unlikely for eggs. If bacterial assemblages on the surface of eggs have a role for their host, it is certainly different from that observed in the cephalothorax of adults. As *R. exoculata* brooding females are found in dense aggregates close to hydrothermal emissions, bacterial assemblages could potentially offer protection against toxic compounds. This hypothesis has already been proposed for adults (Jan et al., 2014), and is supported in our results by the increased bacterial coverage observed on eggs more exposed to hydrothermal fluids and by a high sensitivity of egg brood bacterial assemblages to vent fluid composition. Protection against potential pathogens could be another role. Such anti-pathogen protection was suggested for epibiotic bacteria associated with *Homarus americanus* or *Palaemon macrodactylus* embryos, which produce substances inhibiting growth of pathogenic fungi (Gil-Turnes et al., 1989; Gil-Turnes and Fenical, 1992). Bacterial partners producing potent toxins protecting their hosts are also often described on egg surfaces of several species of terrestrial arthropods. These bacteria play an antipredator role for their insect hosts, as demonstrated by laboratory induced aposymbiotic eggs, which appear to experience more fungal infestation or more predation from spiders than eggs with bacterial symbionts (Kellner and Dettner, 1996; Flórez et al., 2017). All these examples stress the protective role of symbiosis in early life stages of arthropods and suggest that similar processes could be taking place in *R. exoculata*.

*Epsilonbacteraeota, Gammaproteobacteria*, and *Zetaproteobacteria* symbiont-related lineages appear able to colonize different surfaces of their host, such as eggs, pleopods (this study) or antennae (Zbinden et al., 2018). This observation resembles the case of *Kiwa puravida* yeti crabs from cold seeps, which have bacterial coverage on several body parts with closely related lineages (Goffredi et al., 2014). This capacity to settle quickly on different types of surfaces shows a strong ability for colonization in deep sea vent for these groups especially for *Epsilonbacteraeota* for which a highly conserved quorum sensing machinery enabling biofilm formation has been retrieved in every lineages (Pérez-Rodriguez et al., 2015). This machinery, in addition to LuxR genes for the *Gammaproteobacteria*, has furthermore been found in *Rimicaris exoculata* cephalothorax epibionts lineages (Le Bloa et al., 2017). Occurrence of *Epsilonbacteraeota, Gammaproteobacteria* and *Zetaproteobacteria* symbiont-related lineages in vent fluids and microbial mats of vent sites where *R. exoculata* also lives (Petersen et al., 2010; Scott et al., 2015) supports the hypothesis of a horizontal transmission mode for these three groups. More information on larval life of *R. exoculata* is needed to assess whether these bacterial lineages maintain a continuous presence or not throughout their host’s life cycle. Intriguingly, a symbiont-related lineage of *Entomoplasmatales* was the sole bacterial group present on egg samples alone. Despite their very low abundance, our metabarcoding results provide a second report of this group on the eggs of *R. exoculata* with a different sequencing technique: Illumina MiSeq versus 454 Pyrosequencing used in Cowart et al. (2017). Considering technical limitations for detecting bacteria occurring at very low abundance with standard FISH, and the absence of a specific probe for this group, our observations failed to localize them precisely. Potential locations could be the egg surface, as for *Gammaproteobacteria* or *Epsilonbacteraeota*, inside eggs and directly associated with the embryos, or only in the mucus matrix. However, *Entomoplasmatales*-related sequences were not detected in any of our egg brood samples, which could be related to insufficient sequencing depth. Occasional contamination of this group on mucus embedded eggs by maternal anal excretion cannot be eliminated, whether or not this corresponds to a true transmission pathway for *Rimicaris* shrimps. Whereas the presence of *Entomoplasmatales* on eggs is not completely confirmed by our work, we do not exclude the possibility of a vertical transmission for this group, especially since *Entomoplasmatales* have never been retrieved from the surrounding environment. This might occur during embryonic development by release of bacteria from the gut of the mother in the mucus coat during or after oviposition, by infection of ovaries during vitellogenesis as proposed by Durand et al. (2015), or later on by trophallaxis between juveniles and adults (Durand et al., 2015). To improve understanding of symbiont acquisition during the life cycle of *R. exoculata*, targeted FISH experiments with specific *Entomoplasmatales* probes are now needed to precisely locate them, first in the adult digestive system and then in other life stages. More investigations on the internal anatomy of *Rimicaris* larvae could also help to determine the possible existence of a hosting organ for this group at this life stage.

## Author Contributions

PM contributed to data acquisition and analysis, wrote the first draft of the manuscript, review and editing of this manuscript. IH-A contributed for electron microscopy data acquisition and conception and design of the study, review and editing of this manuscript. JA contributed for analysis of sequencing data and review corresponding sections of this manuscript. VC-G contributed for data acquisition and methodology for molecular biology work. NG contributed for electron microscopy data acquisition. BS and LA designed, provided, and serviced the PERISCOP device, and supervised the corresponding *in situ* sampling operations. FP and M-AC-B contributed to conception and design of the study, review and editing of the manuscript, and supervision of the project.

## Conflict of Interest Statement

The authors declare that the research was conducted in the absence of any commercial or financial relationships that could be construed as a potential conflict of interest.

## Supplementary Material

FIGURE S1 X-Ray Spectrophotometric analysis (EDX) showing SEM analyzed areas of mineral crusts or mineral deposits for each sample with the corresponding spectrum. For some samples, bare surface areas clear of minerals were also analyzed as control. Analyzed were performed on **(A,B)** late stage eggs, **(C)** late stage pleopods, **(D)** mid stage eggs, **(E)** and mid stage pleopods.

FIGURE S2 Venn diagrams representing the numbers of shared OTUs across all samples. Affiliations of taxa representative of a body structure, vent field, or developmental stage are indicated.

FIGURE S3 Alpha diversity measures of OTU number (Richness) and Inverse Simpson index (Evenness) compared **(A)** between eggs and pleopods, **(B)** between TAG and Snake Pit egg samples, **(C)** between TAG and Snake Pit pleopod samples, **(D)** between developmental stages of egg samples, and **(E)** between developmental stages of the corresponding eggs for pleopod samples. ^∗^ mean that significant differences were statistically supported.

FIGURE S4 Mean relative abundances per categories of 16S rRNA gene sequence reads according to their classification (Silva 132 database). Groups are at the family level for the *Epsilonbacteraeota* phylum and at the class level for other phyla.

FIGURE S5 Ranked LDA scores of the differentially abundant bacterial taxa, with taxa with highest relative abundance at TAG in orange, and taxa with highest relative abundance at Snake Pit in blue for bacterial communities covering **(A)** eggs and **(B)** pleopods of ovigerous *R. exoculata*.

FIGURE S6 Ranked LDA scores of the differentially abundant bacterial taxa, with taxa with highest relative abundance at early stage in yellow, at mid stage in brown and at late stage in purple for bacterial communities covering **(A)** eggs and **(B)** pleopods of ovigerous *R. exoculata.*

FIGURE S7 Additional FISH observations of *Rimicaris exoculata* eggs. Observations were performed on semi-thin sections (2 μm) stained with DAPI (blue) **(A)** Early stage egg hybridized with Eub338-Cy5 and **(B)** Late stage egg co-hybridized with Eub338-Cy5 and Gam42a-Cy5. Colors, Green (*Eubacteria*), Red (*Gammaproteobacteria*), and Yellow (Co-*hybridized probes*).

TABLE S1 List of samples used in this study.

TABLE S2 List of FISH probes used in this study.

TABLE S3 PERMANOVA analysis.
