# Supplementary material for: Is It First the Egg or the Shrimp? – Diversity and Variation in Microbial Communities Colonizing Broods of the Vent Shrimp Rimicaris exoculata During Embryonic Development
Source: Front Microbiol. 2019 Apr 17;10:808. doi: 10.3389/fmicb.2019.00808 (PMC6478704; doi:10.3389/fmicb.2019.00808)

A) BIC-PL10-Peris3-Rimi8: Late stage egg

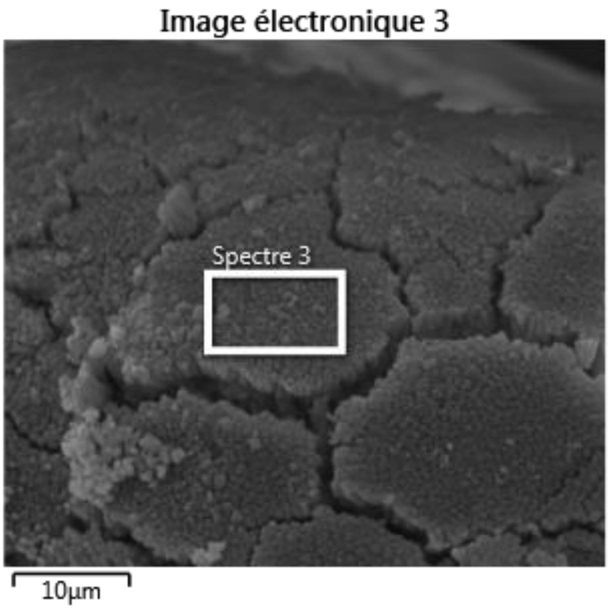

SEM image analyzed by EDX

<u>Spectre 3</u>: **Mineral crust area analyzed for this egg**

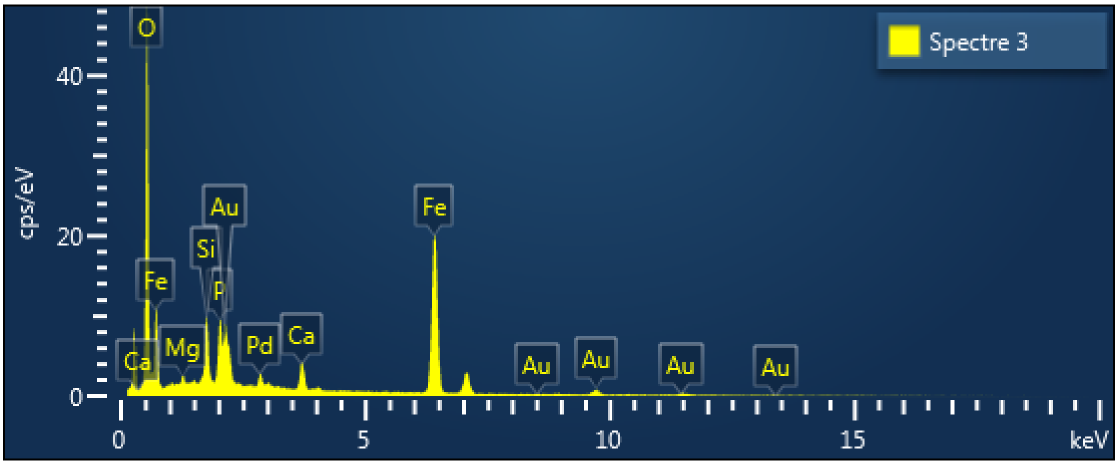

B) BIC-PL08-Peris1-R1: Late stage egg

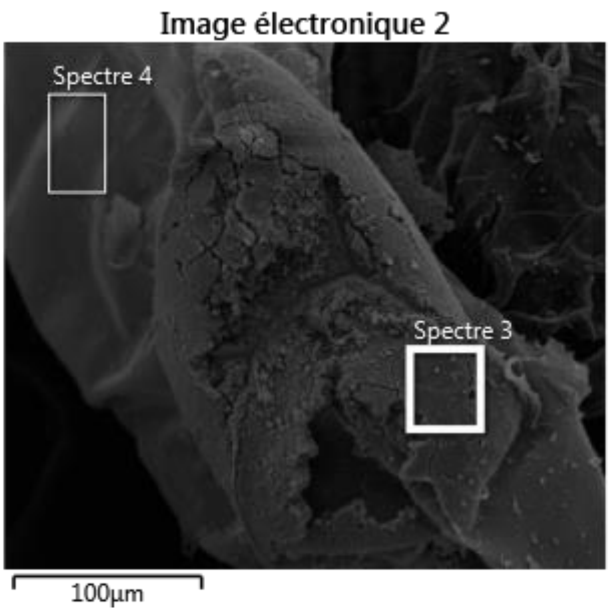

SEM image analyzed by EDX

<u>Spectre 3</u>: Mineral crust area analyzed for this egg

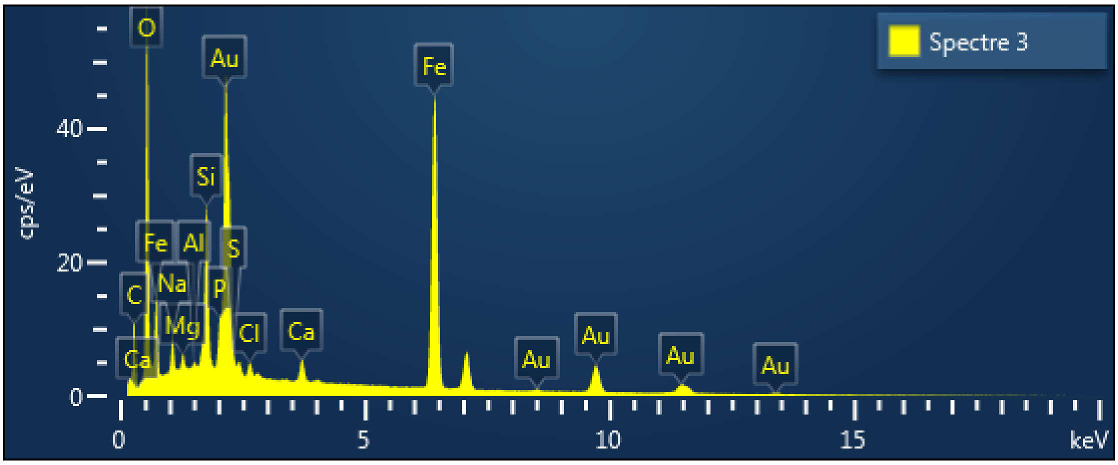

<u>Spectre 4</u>: Bare egg envelope area analyzed for this egg as control

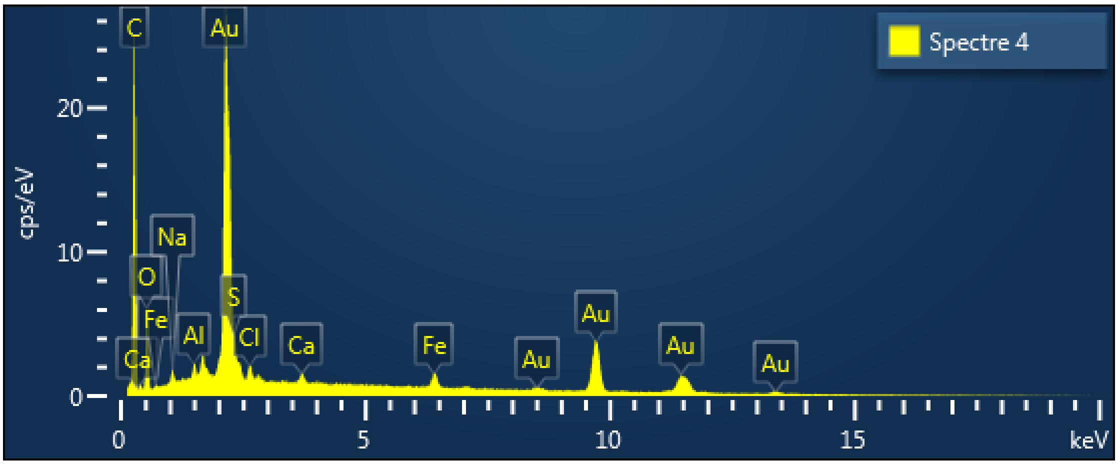

C) BIC-PL08-Peris1-R1: Late stage pleopod

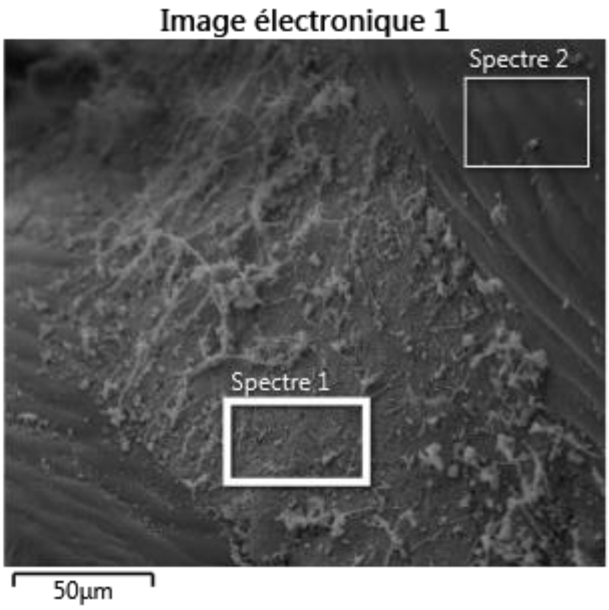

SEM image analyzed by EDX

<u>Spectre 1</u>: **Mineral crust area analyzed for this pleopod**

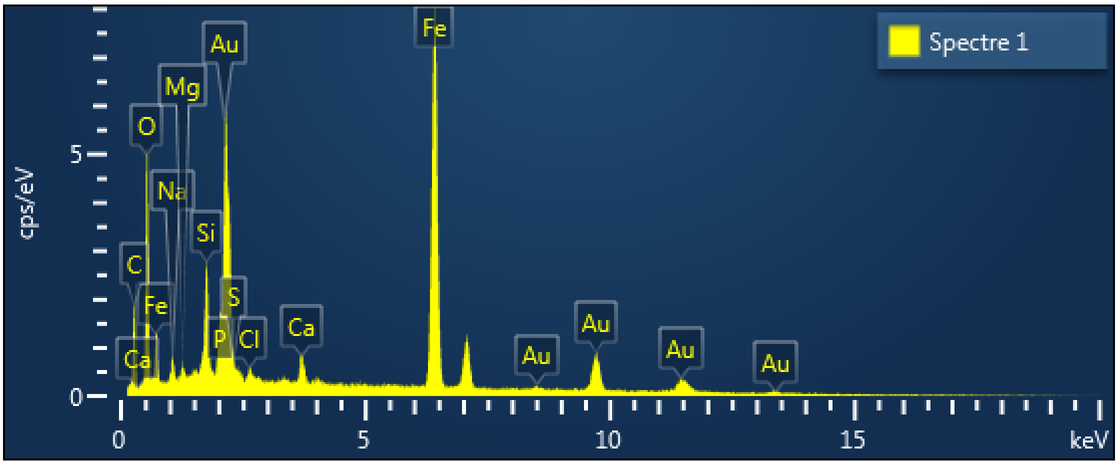

<u>Spectre 2</u>: **Bare pleopod surface area analyzed for this pleopod as control**

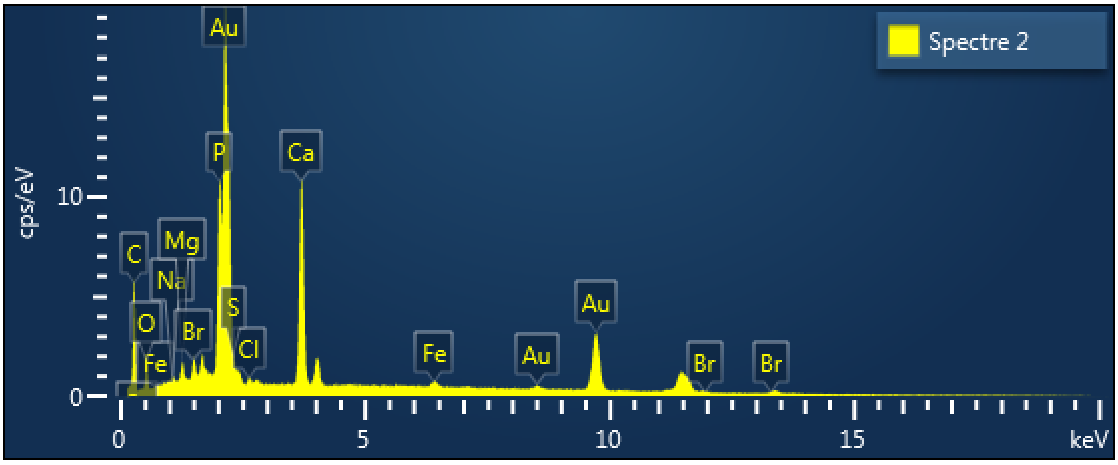

D) BIC-PL12-Peris3-R5: Mid stage egg

Image électronique 4

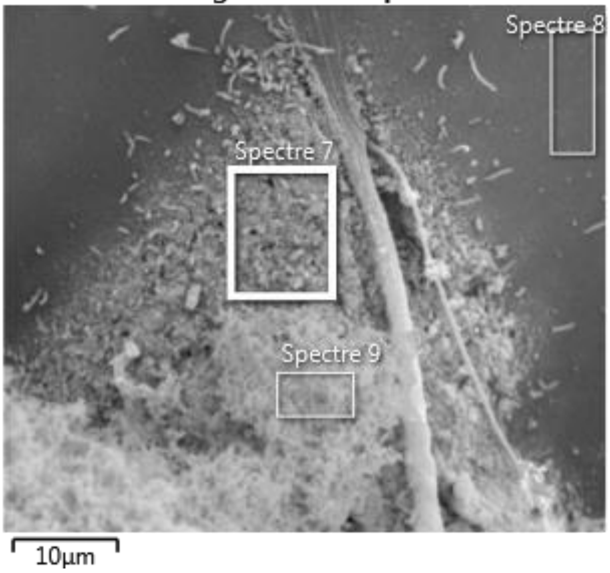

SEM image analyzed by EDX

Spectre 7: Mineral deposit area analyzed for this egg

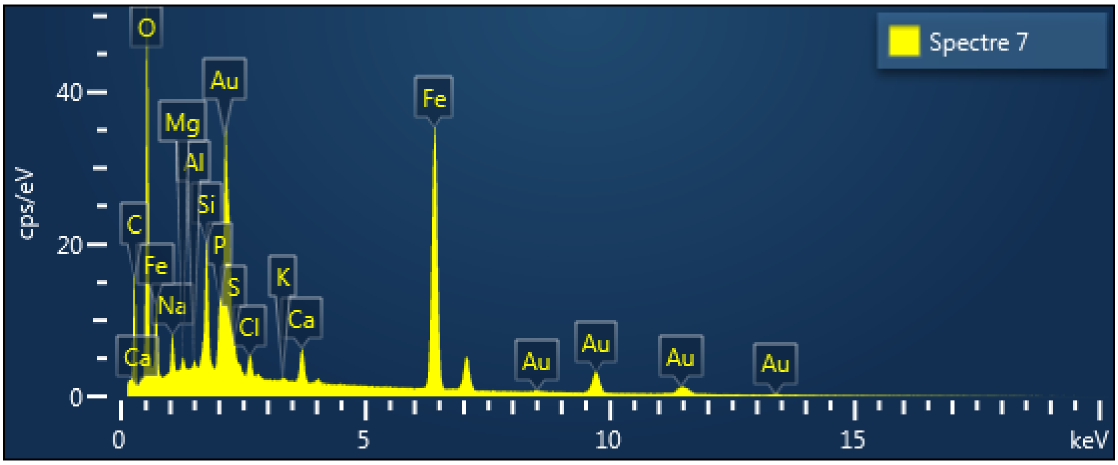

Spectre 8: Bare egg envelope area analyzed for this egg as control

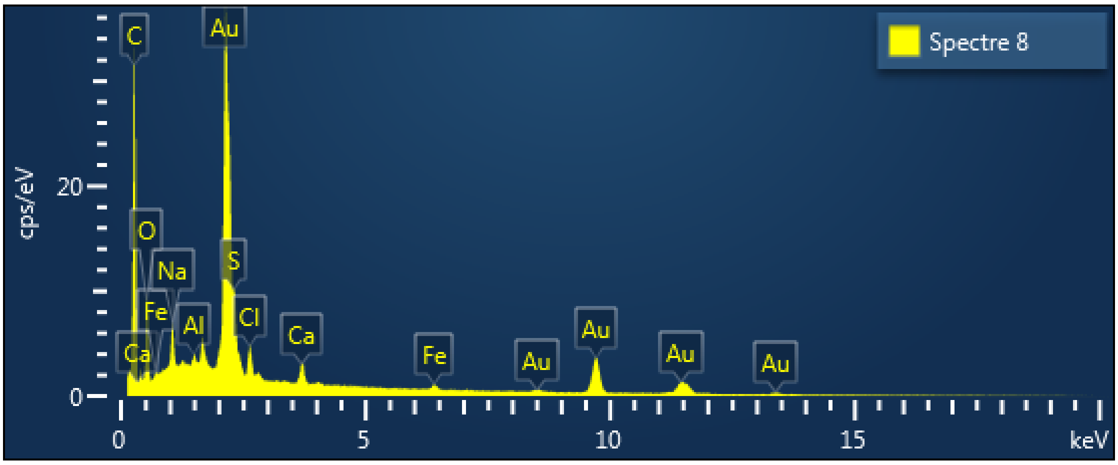

Spectre 9: Additional mineral deposits area analyzed for this egg

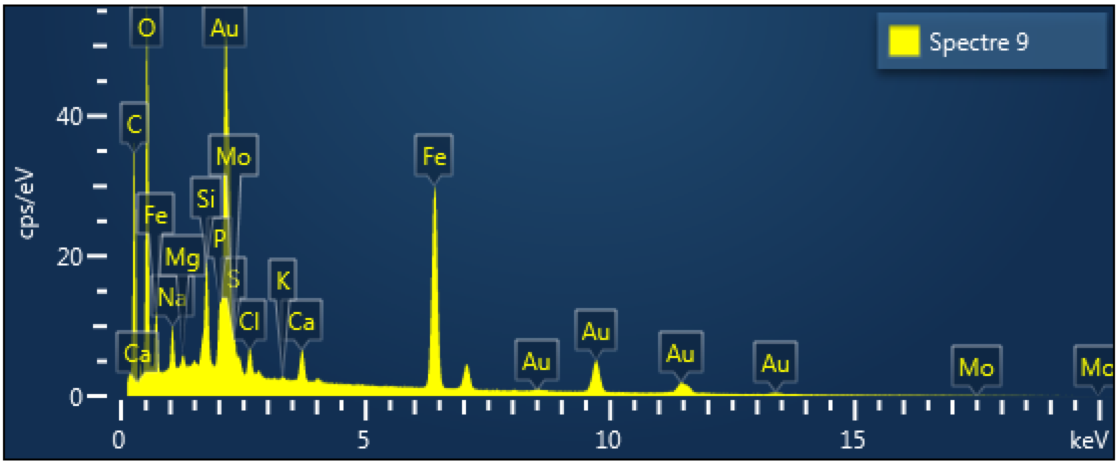

E) BIC-PL12-Peris3-R5: Mid stage pleopod

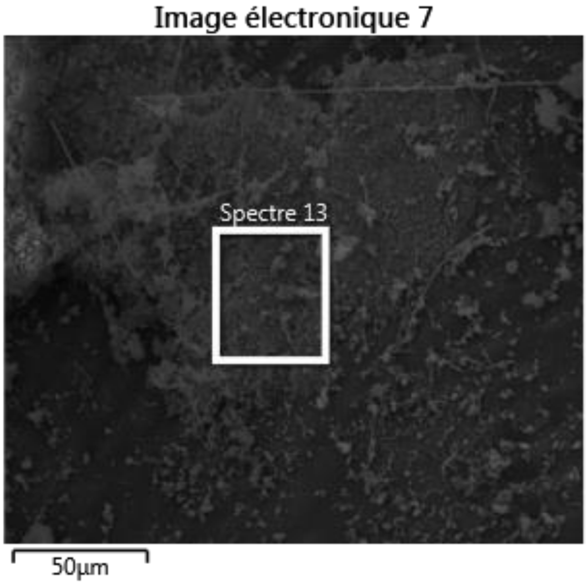

SEM image analyzed by EDX

Spectre 13: Mineral crust area analyzed for this pleopod

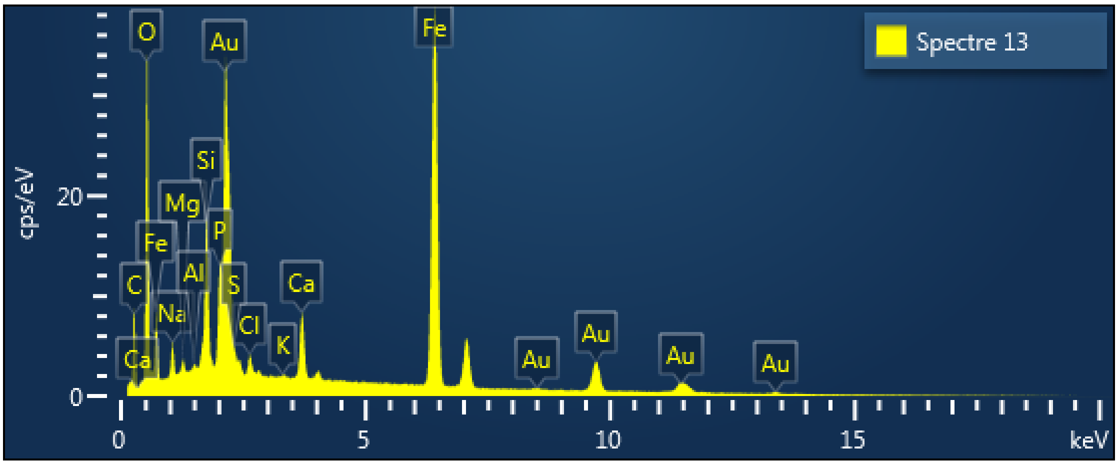

Supplement: FIGURE S1 — X-Ray Spectrophotometric analysis (EDX) showing SEM analyzed areas of mineral crusts or mineral deposits for each sample with the corresponding spectrum. For some samples, bare surface areas clear of minerals were also analyzed as control. Analyzed were performed on (A,B) late stage eggs, (C) late stage pleopods, (D) mid stage eggs, (E) and mid stage pleopods. [file Data_Sheet_1.PDF]
