# Supplementary material for: Is It First the Egg or the Shrimp? – Diversity and Variation in Microbial Communities Colonizing Broods of the Vent Shrimp Rimicaris exoculata During Embryonic Development
Source: Front Microbiol. 2019 Apr 17;10:808. doi: 10.3389/fmicb.2019.00808 (PMC6478704; doi:10.3389/fmicb.2019.00808)
Supplement: FIGURE S2 — Venn diagrams representing the numbers of shared OTUs across all samples. Affiliations of taxa representative of a body structure, vent field, or developmental stage are indicated. [file Data_Sheet_2.PDF]

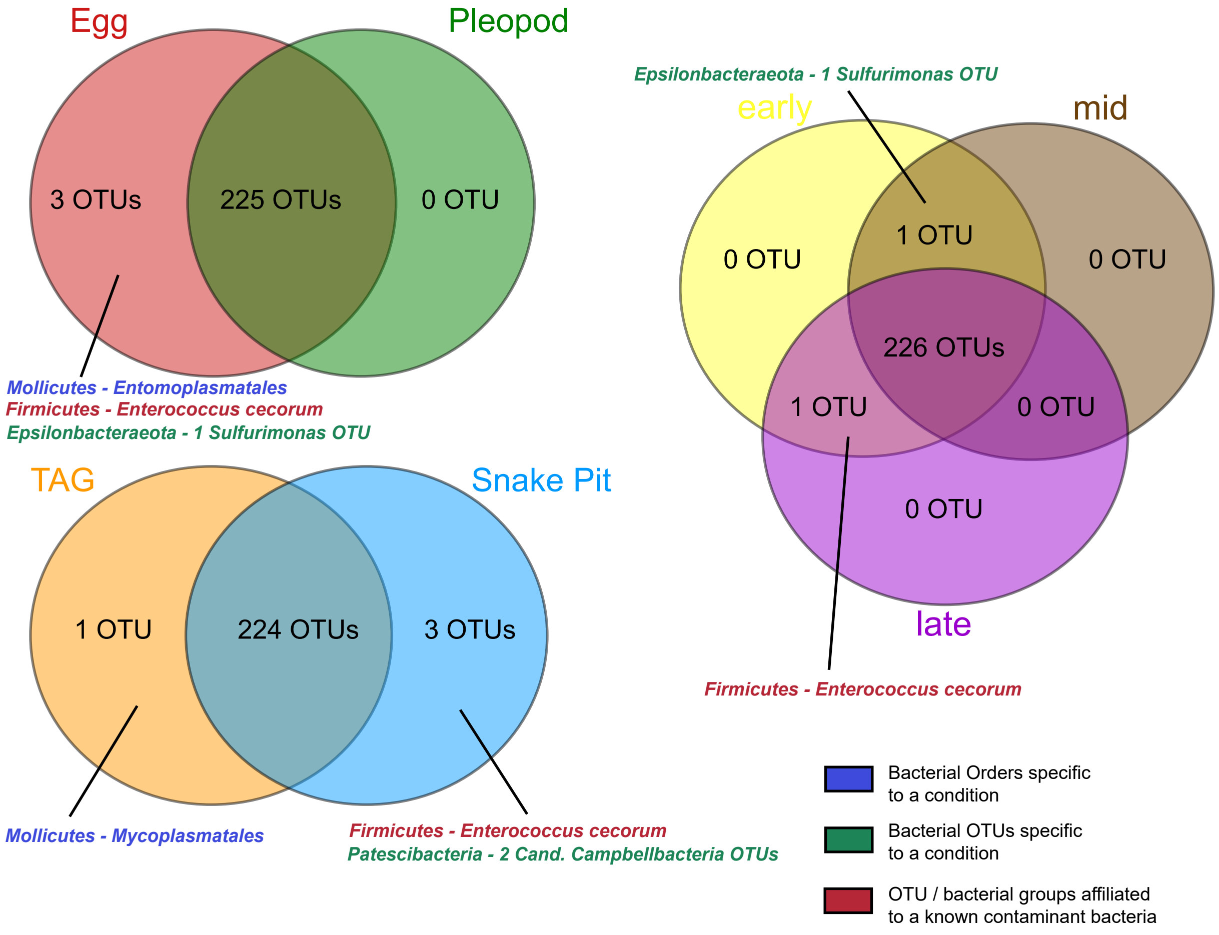

Egg
Pleopod
3 OTUs
225 OTUs
0 OTU
Mollicutes - Entomoplasmatales
Firmicutes - Enterococcus cecorum
Epsilonbacteraeota - 1 Sulfurimonas OTU
Epsilonbacteraeota - 1 Sulfurimonas OTU
early
mid
late
0 OTU
1 OTU
0 OTU
226 OTUs
1 OTU
0 OTU
0 OTU
Firmicutes - Enterococcus cecorum
TAG
Snake Pit
1 OTU
224 OTUs
3 OTUs
Mollicutes - Mycoplasmatales
Firmicutes - Enterococcus cecorum
Patescibacteria - 2 Cand. Campbellbacteria OTUs
Bacterial Orders specific to a condition
Bacterial OTUs specific to a condition
OTU / bacterial groups affiliated to a known contaminant bacteria
