# Supplementary material for: Is It First the Egg or the Shrimp? – Diversity and Variation in Microbial Communities Colonizing Broods of the Vent Shrimp Rimicaris exoculata During Embryonic Development
Source: Front Microbiol. 2019 Apr 17;10:808. doi: 10.3389/fmicb.2019.00808 (PMC6478704; doi:10.3389/fmicb.2019.00808)
Supplement: FIGURE S3 — Alpha diversity measures of OTU number (Richness) and Inverse Simpson index (Evenness) compared (A) between eggs and pleopods, (B) between TAG and Snake Pit egg samples, (C) between TAG and Snake Pit pleopod samples, (D) between developmental stages of egg samples, and (E) between developmental stages of the corresponding eggs for pleopod samples. ∗ mean that significant differences were statistically supported. [file Data_Sheet_3.PDF]

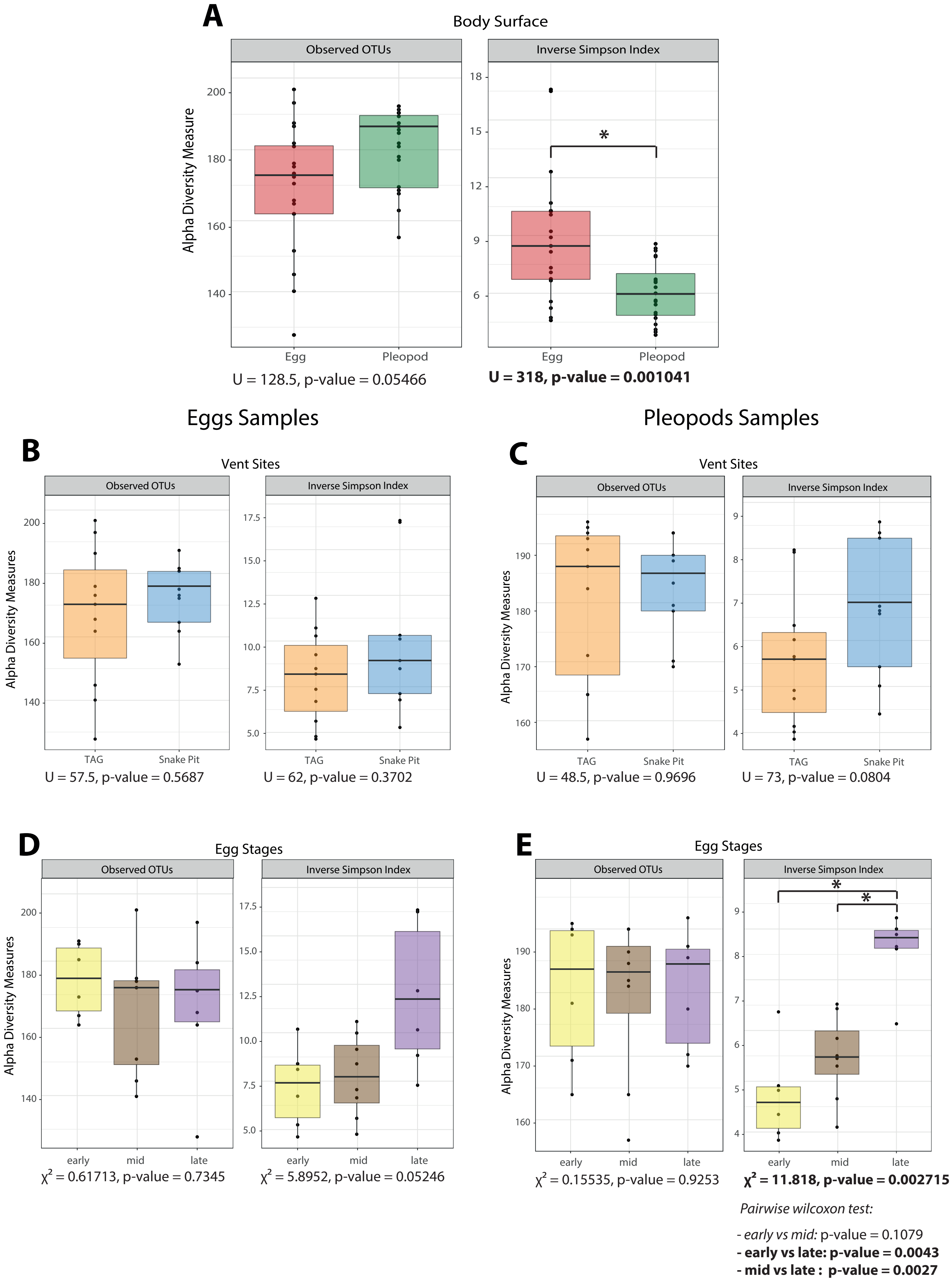
A
Body Surface
Observed OTUs
Inverse Simpson Index
Alpha Diversity Measure
200
180
160
140
18
15
12
9
6
*
Egg
Pleopod
U = 128.5, p-value = 0.05466
U = 318, p-value = 0.001041
Eggs Samples
Pleopods Samples
B
Vent Sites
Observed OTUs
Inverse Simpson Index
Alpha Diversity Measures
17.5
15.0
12.5
10.0
7.5
5.0
TAG
Snake Pit
U = 57.5, p-value = 0.5687
U = 62, p-value = 0.3702
C
Vent Sites
Observed OTUs
Inverse Simpson Index
Alpha Diversity Measures
190
180
170
160
9
8
7
6
5
4
TAG
Snake Pit
U = 48.5, p-value = 0.9696
U = 73, p-value = 0.0804
D
Egg Stages
Observed OTUs
Inverse Simpson Index
Alpha Diversity Measures
early
mid
late
χ2 = 0.61713, p-value = 0.7345
χ2 = 5.8952, p-value = 0.05246
E
Egg Stages
Observed OTUs
Inverse Simpson Index
Alpha Diversity Measures
*
*
early
mid
late
χ2 = 0.15535, p-value = 0.9253
χ2 = 11.818, p-value = 0.002715
Pairwise wilcoxon test:
- early vs mid: p-value = 0.1079
- early vs late: p-value = 0.0043
- mid vs late : p-value = 0.0027
