# Supplementary material for: Is It First the Egg or the Shrimp? – Diversity and Variation in Microbial Communities Colonizing Broods of the Vent Shrimp Rimicaris exoculata During Embryonic Development
Source: Front Microbiol. 2019 Apr 17;10:808. doi: 10.3389/fmicb.2019.00808 (PMC6478704; doi:10.3389/fmicb.2019.00808)
Supplement: FIGURE S4 — Mean relative abundances per categories of 16S rRNA gene sequence reads according to their classification (Silva 132 database). Groups are at the family level for the Epsilonbacteraeota phylum and at the class level for other phyla. [file Data_Sheet_4.PDF]

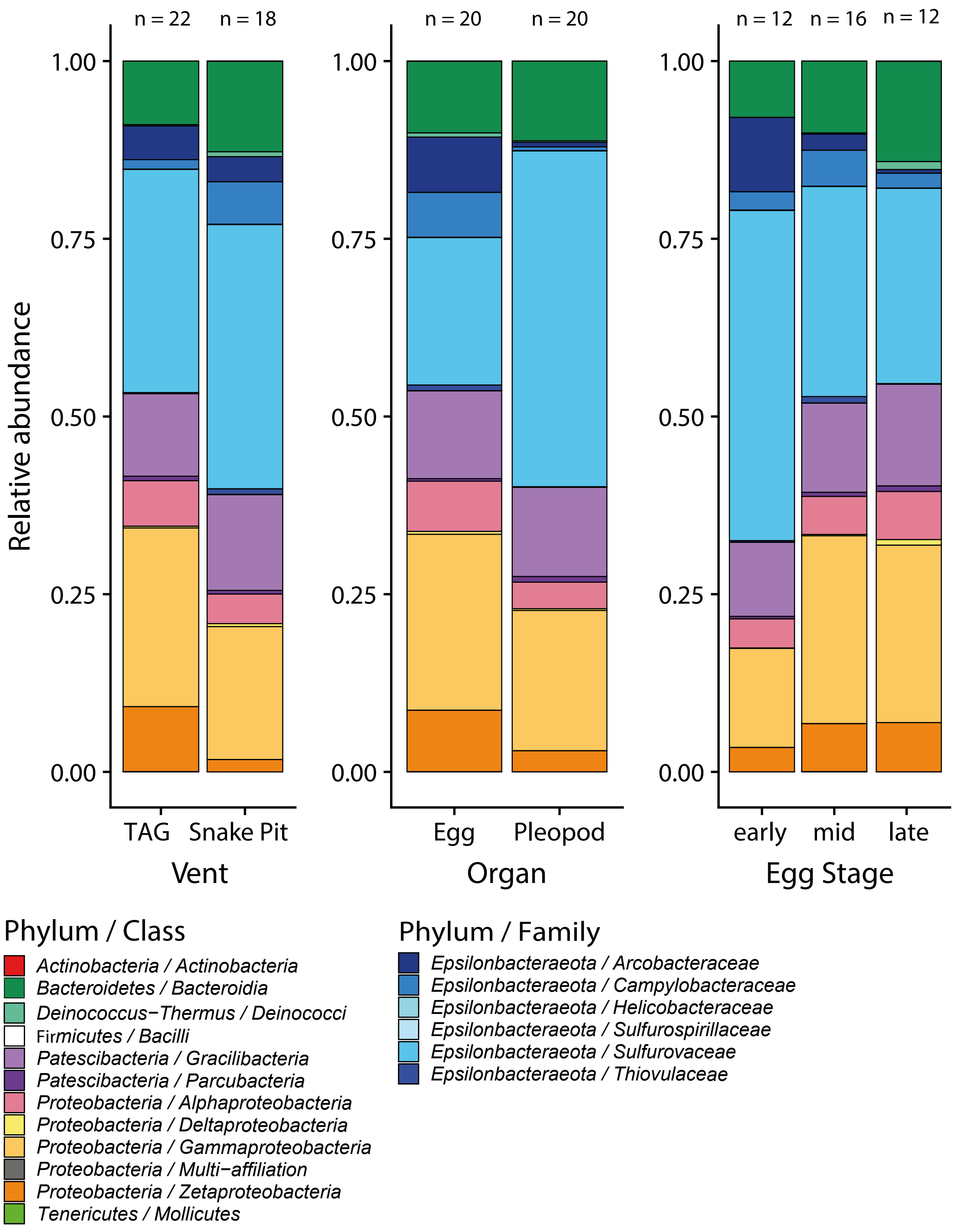

n = 22
n = 18
n = 20
n = 20
n = 12
n = 16
n = 12
Relative abundance
1.00
0.75
0.50
0.25
0.00
TAG
Snake Pit
Vent
Egg
Pleopod
Organ
early
mid
late
Egg Stage
Phylum / Class
Actinobacteria / Actinobacteria
Bacteroidetes / Bacteroidia
Deinococcus−Thermus / Deinococci
Firmicutes / Bacilli
Patescibacteria / Gracilibacteria
Patescibacteria / Parcubacteria
Proteobacteria / Alphaproteobacteria
Proteobacteria / Deltaproteobacteria
Proteobacteria / Gammaproteobacteria
Proteobacteria / Multi−affiliation
Proteobacteria / Zetaproteobacteria
Tenericutes / Mollicutes
Phylum / Family
Epsilonbacteraeota / Arcobacteraceae
Epsilonbacteraeota / Campylobacteraceae
Epsilonbacteraeota / Helicobacteraceae
Epsilonbacteraeota / Sulfurospirillaceae
Epsilonbacteraeota / Sulfurovaceae
Epsilonbacteraeota / Thiovulaceae
