# Supplementary figures and images for: Is It First the Egg or the Shrimp? – Diversity and Variation in Microbial Communities Colonizing Broods of the Vent Shrimp Rimicaris exoculata During Embryonic Development
Source: Front Microbiol. 2019 Apr 17;10:808. doi: 10.3389/fmicb.2019.00808 (PMC6478704; doi:10.3389/fmicb.2019.00808)

A

## Egg Samples

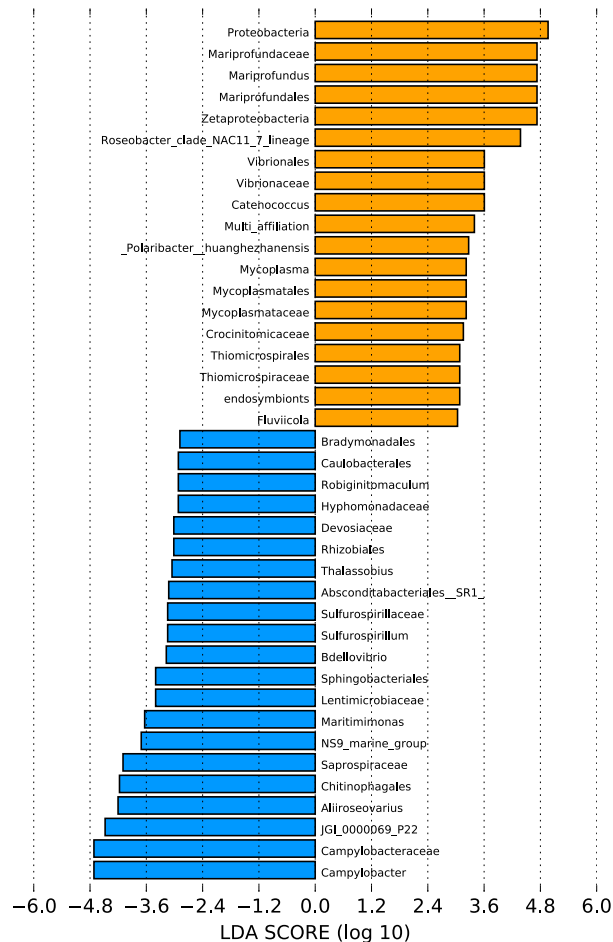

B

## Pleopod Samples

Snake Pit  
TAG

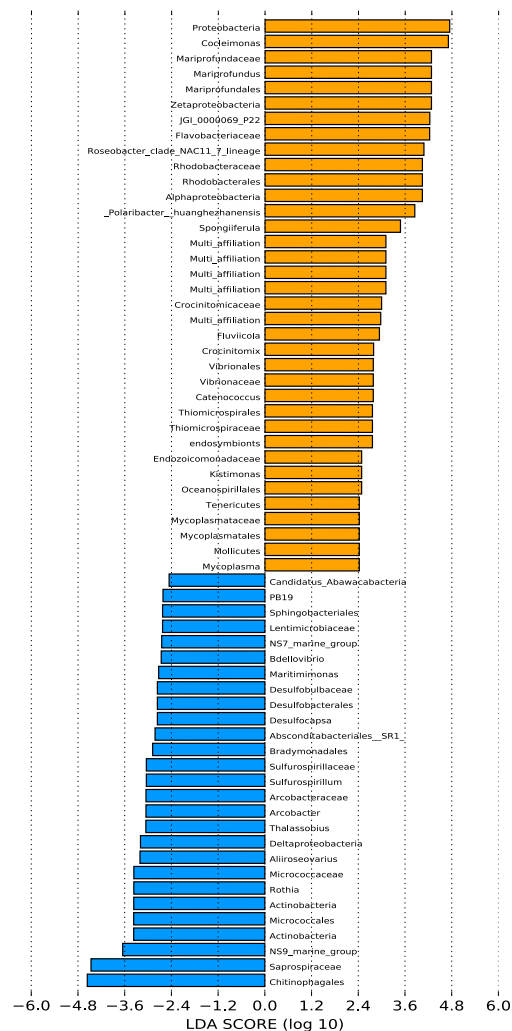

Supplement: FIGURE S5 — Ranked LDA scores of the differentially abundant bacterial taxa, with taxa with highest relative abundance at TAG in orange, and taxa with highest relative abundance at Snake Pit in blue for bacterial communities covering (A) eggs and (B) pleopods of ovigerous R. exoculata. [file Data_Sheet_5.PDF]

A

## Egg Samples

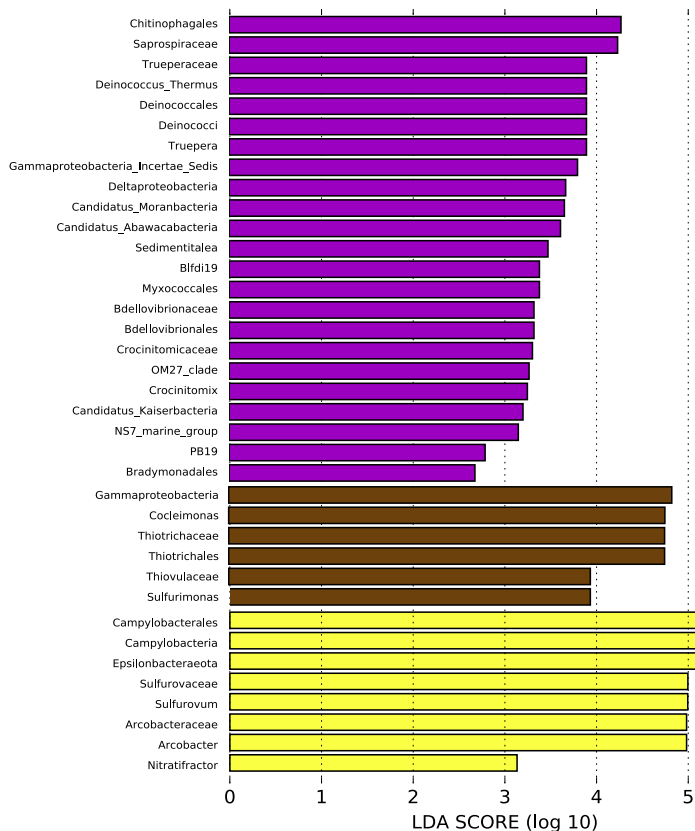

B

## Pleopod Samples

early  
mid  
late

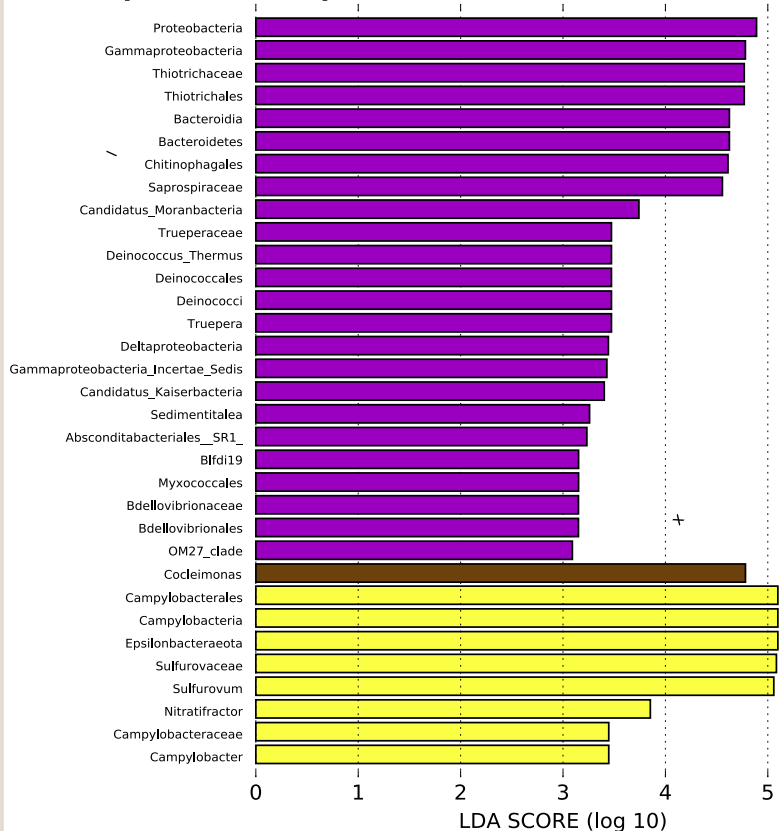

Supplement: FIGURE S6 — Ranked LDA scores of the differentially abundant bacterial taxa, with taxa with highest relative abundance at early stage in yellow, at mid stage in brown and at late stage in purple for bacterial communities covering (A) eggs and (B) pleopods of ovigerous R. exoculata. [file Data_Sheet_6.PDF]

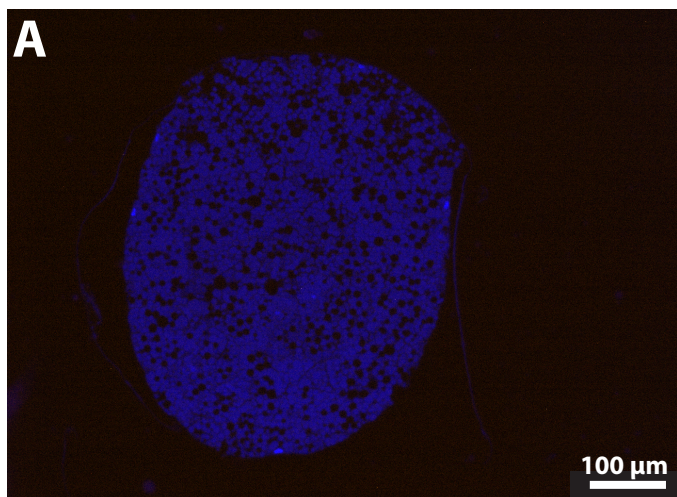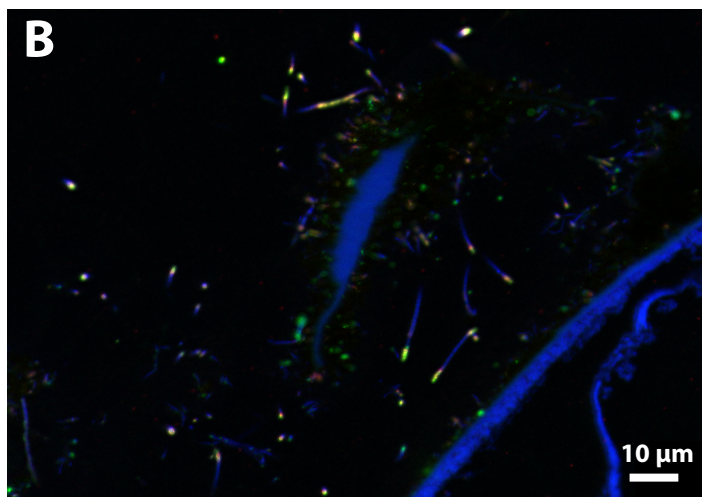

Supplement: FIGURE S7 — Additional FISH observations of Rimicaris exoculata eggs. Observations were performed on semi-thin sections (2 μm) stained with DAPI (blue) (A) Early stage egg hybridized with Eub338-Cy5 and (B) Late stage egg co-hybridized with Eub338-Cy5 and Gam42a-Cy5. Colors, Green (Eubacteria), Red (Gammaproteobacteria), and Yellow (Co-hybridized probes). [file Data_Sheet_7.PDF]
